# Electricity-powered artificial root nodule

**DOI:** 10.1038/s41467-020-15314-9

**Published:** 2020-03-20

**Authors:** Shengtao Lu, Xun Guan, Chong Liu

**Affiliations:** 1 Department of Chemistry and Biochemistry, University of California, Los Angeles, Los Angeles, CA 90095 USA; 2 California NanoSystems Institute (CNSI), University of California, Los Angeles, Los Angeles, CA 90095 USA

**Keywords:** Catalysis, Electrochemistry, Materials chemistry

## Abstract

Root nodules are agricultural-important symbiotic plant-microbe composites in which microorganisms receive energy from plants and reduce dinitrogen (N_2_) into fertilizers. Mimicking root nodules using artificial devices can enable renewable energy-driven fertilizer production. This task is challenging due to the necessity of a microscopic dioxygen (O_2_) concentration gradient, which reconciles anaerobic N_2_ fixation with O_2_-rich atmosphere. Here we report our designed electricity-powered biological|inorganic hybrid system that possesses the function of root nodules. We construct silicon-based microwire array electrodes and replicate the O_2_ gradient of root nodules in the array. The wire array compatibly accommodates N_2_-fixing symbiotic bacteria, which receive energy and reducing equivalents from inorganic catalysts on microwires, and fix N_2_ in the air into biomass and free ammonia. A N_2_ reduction rate up to 6.5 mg N_2_ per gram dry biomass per hour is observed in the device, about two orders of magnitude higher than the natural counterparts.

## Abstract (web summary)

Root nodules are of key importance in nitrogen fixation. Here, the authors report on an electrically powered artificial root nodule for nitrogen fixation made from silicon-based microwires which can accommodate nitrogen fixing symbiotic bacteria for fixing nitrogen into biomass and free ammonia.

## Introduction

**Fig. 1:**
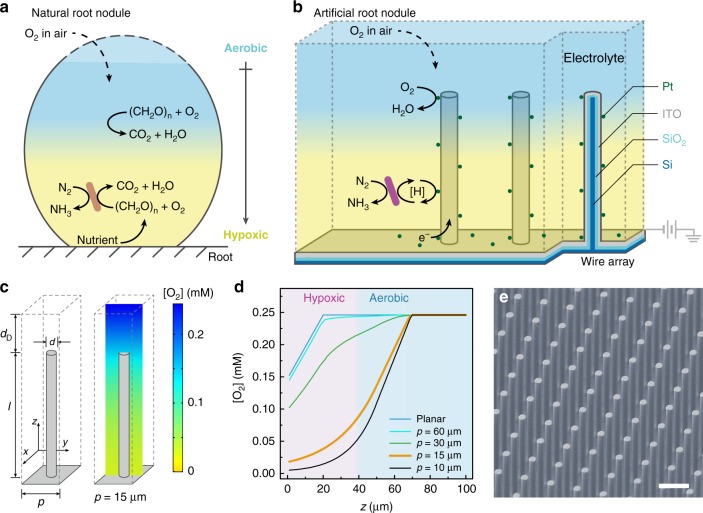
Scheme and the simulated O_2_ gradient of electricity-driven artificial root nodule. Scheme of N_2_ fixation in the natural **a** and the proposed artificial root nodule **b**. **c** Morphology of designed microwire array and the cross-sectional display of simulated O_2_ concentration ([O_2_]) profile. Length (*l*), 50 μm; diameter (*d*), 4 μm; periodicity (*p*), 15 μm; diffusion layer thickness (*d*_D_), 20 μm. **d** Simulated [O_2_] against the distance from the base of electrode (*z*) for planar electrode and microwire arrays of different *p* values (*l* = 50 μm, *d* = 4 μm). The hypoxic (pink) and aerobic (blue) domains for wire array of *p* = 15 µm are suggested. **e** Scanning electron microscopy (SEM) image of the prepared Si wire array with a morphology shown in **c**. ITO indium-tin oxide. Scale bar, 15 μm. Electrochemical potential (*E*_appl_) = 0.5 V vs. reversible hydrogen electrode (RHE) in **c** and **d**.

A major component of global dinitrogen (N_2_) fixation occurs in root nodules^1^, the symbiotic plant–microbe composites in which microorganisms receive energy from plants and reduce N_2_ into fertilizers^2–4^. The significance of biological root nodules in agriculture inspires us to consider a man-made mimic, which houses diazotrophs^5,6^ and fixes N_2_ in air with renewable energy such as solar electricity. Biological|inorganic hybrid systems are reported to use sustainable energy, such as light and renewable electricity to drive biochemical reactions with high throughput and energy efficiency^7–11^. We recently reported a biocompatible system^7^ that utilizes electricity to power the N_2_ fixation in *Xanthobacter autotrophicus*^12^, a non-symbiotic root-associative diazotroph^13^. In this system, water is split into O_2_ and H_2_ with small thermodynamic driving forces^12^. The generated H_2_ as a reducing equivalent is selectively consumed by the hydrogenases in autotrophic microorganisms and powers the biochemical reduction of carbon dioxide (CO_2_) and N_2_^12^. However, this hybrid system operates at a microaerobic condition of 2% O_2_ other than ambient air, which limits its potential for practical applications. In root nodules, the anaerobic nature of N_2_-fixing nitrogenases is incompatible with the aerobic metabolism in plant cells^14,15^. In order to accommodate this incompatibility for a successful N_2_ reduction in air, the O_2_ environment in root nodules quickly changes from aerobic at the apex to hypoxic towards the base across tens or hundreds of micrometers (Fig. 1a)^16–18^. Such a steep O_2_ gradient in living organisms has been challenging to mimic with a man-made device^19^. Designs of hydrogel and microfluidic systems have been employed to create steady-state concentration profiles, yet these delicate setups require a continuous gas purging or supply of O_2_ scavengers, which are not readily tunable or attainable with long-term stability^19–21^. While biofilms hosting diazotrophs have been recently reported to exhibit N_2_ fixation in air^22^, their complex and less-well-defined nature renders certain challenges (Supplementary Note 1).

Here we report our strategy that uses microwire array electrode to create a controllable O_2_ gradient for the construction of an electricity-driven artificial root nodule (Fig. 1b). The wire array compatibly accommodates N_2_-fixing symbiotic bacteria within it and provides energy and reducing equivalents to the bacteria. Resembling the natural root nodules, the generated local hypoxic environment within the microwire array enables the biological N_2_-fixation in air. The reported biological|inorganic hybrid system can potentially supplement non-legume crops with electricity-generated fertilizers and reduce the reliance on synthetic alternatives.

## Results

### Design principle and numerical simulations

In our design, microwire array electrode provides a suitable platform of delivering reducing equivalents to biocatalysts, as well as establishing a controllable O_2_ environment for biological N_2_ fixation in air (Fig. 1b). Under a cathodic electrochemical potential, the large surface area of wire array electrode offers fast transfer of reducing equivalents to the microbes through either a direct or a mediated pathway (Fig. 1b)^9,23^. Moreover, in air, the electrochemical reduction of O_2_ on electrode’s surface consumes O_2_ and creates a local O_2_ gradient in the solution. This effect is much more pronounced for porous and large-surface-area electrodes in general^24–27^, effectively creating an O_2_-free domain within the wire array suitable for anaerobic N_2_ fixation (Fig. 1b). Our previous work demonstrated a O_2_-free domain within a nanowire array electrode^23,25^, which allows anaerobic microbial reduction of CO_2_ in air^23^. Here we apply the same design principle for an artificial root nodule. Numerical simulations based on a microkinetic model were applied to design a suitable wire array geometry for biological N_2_ fixation (see “Methods” section). When the wire electrode was at an electrochemical potential (*E*_appl_) cathodic enough to reduce O_2_ (*E*_appl_ = 0.5 V vs. reversible hydrogen electrode, RHE) at pH = 7.0 (same below), the O_2_ concentration near the electrode ([O_2_]) quickly decreases towards the base of wire array (Fig. 1c), establishing an O_2_ gradient more significant than the case when a planar electrode is employed (Fig. 1d and Supplementary Fig. 1). We studied how the periodicity of wire array (*p*) impacts the constructed O_2_ gradients. Larger value of *p* leads to a gentler O_2_ gradient (Fig. 1d), illustrating the tunability of constructing O_2_ gradient with different *p* values. At *p* = 15 µm and wire length *l* = 50 µm (yellow trace in Fig. 1d), a majority of the solution within the wire array is predicted to be hypoxic (<5% O_2_, pink area in Fig. 1d)^19^, suitable for biological N_2_ fixation (see Supplementary Note 2)^5,12^.

### Fabrication and characterization of wire array electrodes

The microwire array device, designed based on numerical simulation (Fig. 1c, d), was constructed for electricity-driven N_2_ fixation in air. A five-step fabrication process (see “Methods” section) led to a core–shell microwire array, which contains a silicon (Si) microwire core, a SiO_2_ insulating coating, a layer of indium-tin oxide (ITO), and Pt particles deposited via sputtering (Fig. 1b)^28^. The electrically conductive ITO layer ensures a uniform distribution of *E*_appl_ on wires’ surface; the Pt particles not only transfer reducing equivalents to diazotrophs for N_2_ fixation but also electrochemically consume O_2_ for a controlled O_2_ gradient^29,30^. The morphology and composition of the wire array were characterized and confirmed by scanning electron microscopy (SEM) equipped with energy-dispersive X-ray spectroscopy (EDS) (Fig. 1e and Supplementary Fig. 2). Linear scan voltammograms (LSVs) of the array electrode in an all-inorganic medium for diazotrophs displayed an onset potential of O_2_ reduction at 0.8 V vs. RHE and a noticeable generation of H_2_ when *E*_appl_ < 0 V vs. RHE (Supplementary Fig. 3 and see “Methods” section), confirming the proposed functionalities of deposited Pt catalysts.

### Optical quantification of O_2_ gradient within wire array

**Fig. 2:**
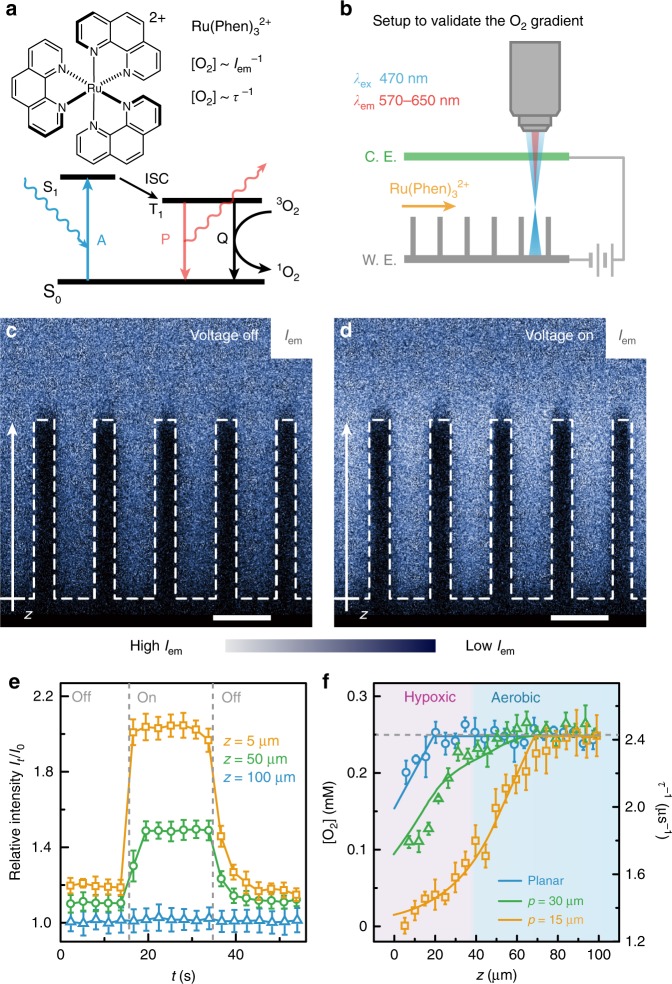
Experimental validation of microscopic O_2_ gradient in microwire array. **a** Simplified Jablonski diagram of Ru(phen)_3_^2+^ including the absorption (A), intersystem crossing (ISC), phosphorescence (P), and the quenching (Q) of its triplet excited states by O_2_. *I*_em_ and τ denote the intensity and lifetime of phosphorescence emission, respectively. **b** Electrochemical setup under a confocal microscope that in situ maps O_2_ gradient. WE denotes microwire array working electrode and CE denotes counter electrode. The setup is mounted on an inverted microscope so the wire array is facing down during observation (as shown in Supplementary Fig. 4). **c**, **d** Cross-sectional *I*_em_ profiles in wire array in the absence and presence of *E*_appl_, respectively. Dashed lines depict wire morphology. Blue pseudo-colored. Scale bars, 15 μm. **e** The temporal response of relative intensities *I*_em_ (*I*_t_/*I*_0_) at different *z* values within wire array. *E*_appl_ was switched on and off at *t* *=* 15 and 35 s, respectively (*n* = 3). **f** Simulated (lines) and experimentally determined (points, *n* = 3) [O_2_] profiles at different electrode geometries. Unless specified, all results, as well as the suggested hypoxic (pink) and aerobic (blue) areas, refer to the geometry shown in Fig. 1c under *E*_appl_ = 0.5 V vs. RHE. Error bars = standard deviation (*n* = 3).

We experimentally validated the predicated O_2_ profile within the designed wire array using confocal microscopy. Tris(1,10-phenanthroline) ruthenium(II) dication, Ru(phen)_3_^2+^, was employed as a molecular O_2_ sensor. After optical excitation (*λ*_ex_ = 470 nm) and a subsequent intersystem crossing (ISC), the photo-excited triplet state (T_1_) of Ru(phen)_3_^2+^ can be effectively quenched by O_2_ in the solution (Fig. 2a)^31,32^. Thus the intensity (*I*_em_) and lifetime (*τ*) of phosphorescence emission for Ru(phen)_3_^2+^ are inversely proportional to local [O_2_], a relationship that allows optical mapping of [O_2_] profile. We constructed an electrochemical set-up that houses the wire array in a three-electrode configuration on a confocal microscope (see “Methods” section, Fig. 2b and Supplementary Fig. 4). Under a constant flow of aerated medium solution of 0.1 mM Ru(phen)_3_^2+^, the spatial distribution of *I*_em_, subsequently the local [O_2_] near the electrode, was characterized at different *E*_appl_ and microwire morphologies (Fig. 2c, d and Supplementary Fig. 5). The side-views of three-dimensional *I*_em_ mappings were displayed when *E*_appl_ was absent (“voltage off”) in Fig. 2c and *E*_appl_ = 0.5 V vs. RHE (“voltage on”) in Fig. 2d. The *I*_em_ profile in Fig. 2d, qualitatively stronger than the one in Fig. 2c, suggests a local O_2_ depletion in the wire array. Temporal control of the established O_2_ gradient was achieved, as the normalized values of *I*_em_ (*I*_t_/*I*_0_) at different depths of the wire array were all responsive towards *E*_appl_ variations (Fig. 2e). It is worth noting that Fig. 2c, d are just qualitatively illustrating the presence of O_2_ gradient, since the light absorption of Si microwire may interfere for a quantitative analysis of O_2_ concentrations based on phosphorescent intensity. In order to circumvent such an issue, we applied phosphorescence lifetime imaging microscopy (PLIM) technique to quantify the O_2_ gradient (Fig. 2f) after establishing a calibration curve (Supplementary Fig. 6). Figure 2f displayed the axial [O_2_] profiles (*z*-direction) of planar (blue points) and wire arrays with *p* = 15 and 30 µm (yellow and green points, respectively), along with the predicted [O_2_] profiles based on numerical simulations (solid lines). The high fidelity between experimental and simulation results illustrates the viability of controlling microscopic O_2_ gradient by using microwire array electrode. As predicted by simulation, a hypoxic domain mimicking the zone of N_2_ fixation in root nodules (pink area in Fig. 2f) was fulfilled.

### Electricity-driven N_2_ reduction with microbes in wire array

**Fig. 3:**
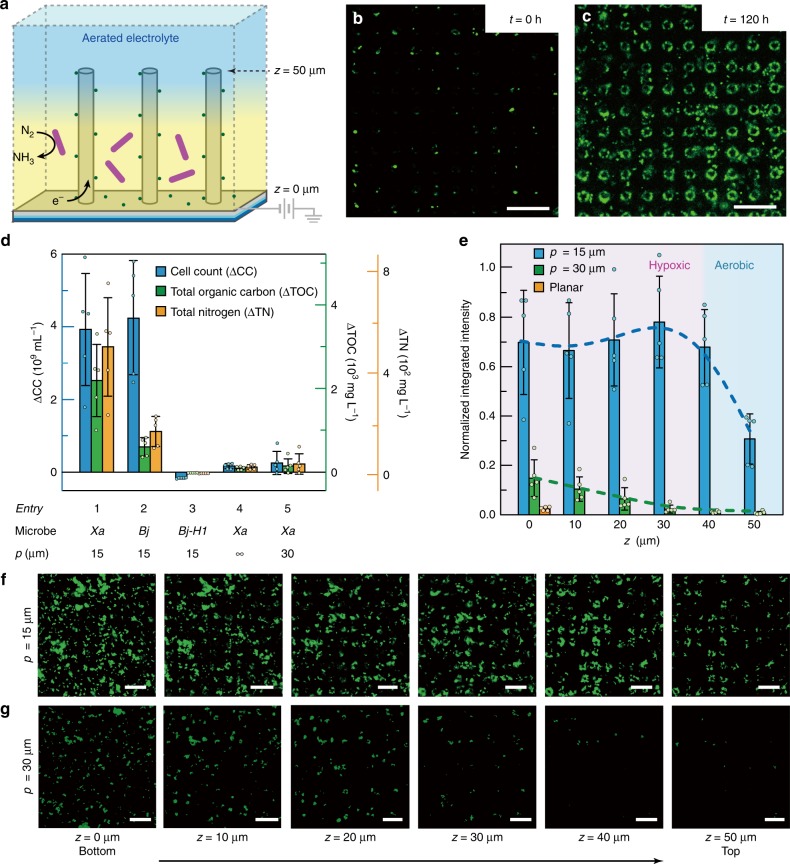
Electricity-driven N_2_ fixation in air. **a** Schematic illustration of the functional device. **b**, **c** Fluorescent images of *B. japonicum*, indicative of biomass accumulation, before **b** and after **c** 120-h operation in microwire array. *E*_appl_ = −0.15 V vs. RHE, *p* = 15 µm, images taken at *z* = 20 μm. **d** Changes of cell count (ΔCC), as well as the retained total nitrogen (ΔTN) and total organic carbon (ΔTOC) in biomass in the device, calculated as the differences between values before and after 120-h operation with the provision of electricity (*n* = 5). ∞, planar electrode; *Xa* *X. autotrophicus*; *Bj B. japonicum, Bj-H1* *B. japniucum-H1*, a *B. japonicum* variant with disrupted *nifH* gene. **e** Integrated fluorescent intensities of *X. autotrophicus*, indicative of biomass accumulation, at different *z* positions for different electrode morphologies. The hypoxic (pink) and aerobic (blue) domains for wire array of *p* = 15 µm are suggested (*n* = 5). **f**, **g** Series of *x–y* cross-sectional fluorescent images at different *z* planes for *X. autotrophicus* population within wire arrays of *p* = 15 μm **f** and *p* = 30 μm **g**. Pseudo-colored in green. Scale bars, 30 μm. The setup for imaging is mounted on an inverted microscope so the wire array is facing down during observation (as shown in Supplementary Fig. 4). For panels **d** and **e**, dot-plot of individual measurements is overlaid with the bar graph. Error bars = standard deviation (*n* = 5).

Electricity-driven N_2_ reduction in air was demonstrated when inoculating N_2_-fixing soil microorganisms into the pre-designed microwire array. *X. autotrophicus* (*X. autotrophicus*, ATCC 35674)^12,13^ and *Bradyrhizobium japonicum* (*B. japonicum*, USDA DES 122), a soybean symbiont model strain capable of oxidizing H_2_ as the energy source^5,33^, were inoculated separately into microwire array electrodes shown in Fig. 1c. In an air-equilibrated liquid medium deprived of any nitrogen and organic carbon sources (see “Methods” section), an electrochemical potential (*E*_appl_ = −0.15 V vs. RHE) was supplied to establish the proposed O_2_ gradient and deliver reducing equivalents, possibly H_2_, for biological N_2_ fixation (Fig. 3a). In 120 h after inoculation, confocal fluorescence microscopy images (see “Methods” section) indicate significant increase of microbial population and biomass for both *B. japonicum* and *X. autotrophicus* strains (Fig. 3b, c and Supplementary Fig. 7). As the only possible source of nitrogen and carbon in the biomass is the N_2_ in the air and CO_2_/bicarbonate in the medium, such a biomass increase suggests biological N_2_ and CO_2_ fixation. In contrast, when we inoculated a *B. japonicum* variant unable to fix N_2_ with its disrupted *nifH* gene (*B. japonicum-H1*)^34,35^, negligible biomass accumulation was observed under the same condition (Supplementary Fig. 8). This indicates that the observed biomass accumulation is concurrent with the expression of nifH protein essential to N_2_ reduction. Last, when there was not enough driving force (*E*_appl_) from electricity to drive N_2_ fixation, no net accumulation of biomass was observed (Supplementary Fig. 9). It supports our claim that electricity is the sole energy source of N_2_ fixation.

After 120 h since inoculation, quantitative assays (see “Methods” section) show that the changes of microbial cell counts (ΔCC) within the wire array were 4.0 ± 1.5 × 10^9^ and 4.2 ± 1.6 × 10^9^ cells mL^−1^ for *X. autotrophicus* and *B. japonicum* strains, respectively (entries 1 and 2 in Fig. 3d; *n* = 5, ± denotes standard deviation, same below). The final microbial cell counts in the device are 19 and 2.5 times of the values right after the inoculation (2.2 ± 1.2 × 10^8^ and 2.7 ± 1.8 × 10^9^ cells mL^−1^) for *B. japonicum* and *X. autotrophicus*, respectively. This highlights that the developed system is biocompatible even for symbiotic microorganisms that might demand additional stimuli for N_2_ fixation (Supplementary Note 3)^4,36^. In correspondence to the change of cell counts, the changes of total nitrogen content (ΔTN) retained in biomass in the device, an indicator of biological N_2_ fixation, were 5.7 ± 2.3 × 10^2^ and 1.9 ± 0.7 × 10^2^ mg L^−1^ for *X. autotrophicus* and *B. japonicum* strains, respectively; the changes of total organic carbon (ΔTOC), another indicator of microbial biomass accumulation, were 2.5 ± 1.0 × 10^3^ and 6.9 ± 2.5 × 10^2^ mg L^−1^ for *X. autotrophicus* and *B. japonicum* strains, respectively. Moreover, we observed the accumulation of free ammonia in the effluent out of the electricity-driven N_2_ fixation device. When 20 mL of medium was circulated throughout the device during a 120-h application of electricity, the nitrogen content in terms of ammonia in the liquid effluent was measured to increase by 0.017 ± 0.003 mg L^−1^ for *B. japonicum*; no detectable free ammonia was observed for *X. autotrophicus* strain under the same experimental condition, consistent with our prior report with *X. autotrophicus* strains in the absence of any external inhibitor^12^ (see “Methods” section). This implies that under a flow condition part of the fixed ammonia is capable to passively diffuse extracellularly, resembling the scenario in the symbiotic system within a root nodule^37^. As far as we know, microbial-based free ammonia production from electricity has not yet been reported except our prior work that utilized inhibitor to purposely prevent microbes from up-taking ammonia^12^, a non-ideal practice for long-term operation. Overall, the electricity-driven artificial root nodule is capable of fixing N_2_ in air at a rate of 6.5 and 1.1 mg N_2_/g dry biomass per hour for *B. japonicum* and *X. autotrophicus* strains, respectively (see “Methods” section). Such rates of N_2_ fixation in our system are about two orders of magnitudes higher than their natural counterparts in the symbiotic root nodules with higher energy efficiencies (see “Methods” section, Supplementary Table 1 and Supplementary Note 4)^4,38^.

### O_2_ gradient within the wire array is crucial to N_2_ fixation

The established O_2_ gradient within the wire array is crucial to the functionality of biological N_2_ fixation in air. Wire array in the absence of deposited Pt particles did not lead to observable N_2_ fixation (Supplementary Fig. 9). It suggests that Pt particles are effective towards oxygen reduction, enable the establishment of O_2_ gradient, and deliver reducing equivalents to the microbes via reducing protons or possibly other direct routes of charge transfer (Supplementary Note 5). Moreover, the microbial distribution at different depths of the wire array (Fig. 3e), characterized by confocal microscopy, suggests a strong correlation between the O_2_ gradient and the microbial population density. When planar electrode or wire array of *p* = 30 µm were inoculated with diazotrophs (entries 4 and 5 in Fig. 3d, respectively), minimal N_2_ fixation was observed. While the wire array of *p* = 15 µm exhibits high microbial density until the very top (*z* > 40 µm in Fig. 3e), which is in accordance with the O_2_ gradient measured under the same *E*_appl_ for nitrogen fixation experiment (Supplementary Note 6 and Supplementary Fig. 10), planar substrate or wire array of larger *p* value (*p* = 30 µm) did not display significant microbial accumulation. Such a difference is also visible in Fig. 3f (*p* = 15 µm) and Fig. 3g (*p* = 30 µm), which highlight the active diazotrophs at different depths of wire arrays. These results illustrate that microwire array not only delivers reducing equivalents for N_2_ fixation, but also creates the hypoxic domains in air as the root nodules do.

## Discussion

Designing wire array electrodes provide a unique and viable approach to program O_2_ gradient at microscopic length scale with high fidelity. Our reported proof-of-concept demonstrates that the established O_2_ gradient enabled by micro-structured electrodes allows the production of nitrogen fertilizer by O_2_-sensitive microbes in air using sustainable electricity (Supplementary Fig. 11), replacing the carbohydrates from natural photosynthesis as the driving force for biological N_2_ fixation. The observed generation of free ammonia from symbiotic microorganisms driven by electricity, differs from prior result of planktonic species^12^, highlights the unique biocatalytic utilities of symbiotic diazotrophs in the future. Our demonstration also offers the design principle of porous electrodes^24^ (Supplementary Note 7) for future N_2_-fxing variants that only contains biocompatible earth-abundant materials (Supplementary Note 8), provides lower cost and better scalability with ingenious engineering design, and employs genetically modified microbes with higher intrinsic rates of N_2_ fixation. In the long run, the concept of biological|inorganic hybrid system will potentially supplement non-legume crops with electricity-generated fertilizers and reduce the reliance on synthetic alternatives.

## Methods

### Chemicals and materials

The chemicals, unless otherwise specified, were purchased from Sigma-Aldrich and used as received. The ITO-coated glass slides were obtained from SPI supplies. Silicon wafers were purchased from University Wafer. Silver paint was purchased from Ted Pella. Chemical-resistant tapes were purchased from McMaster-Carr. Plastic tubing and fittings were obtained from IDEX Health & Science. Polystyrene plates were purchased from Evergreen Scale Models. A-B epoxy adhesives were obtained from Amazon.

### Numeric simulation of O_2_ profile

A three-dimensional microkinetic model in aqueous solution was constructed and simulated using COMSOL Multiphysics (Ver. 5.3). The morphology of the simulated electrode is shown in Fig. 1c. A periodic boundary condition was applied in the *x* and *y* directions (Fig. 1c). A diffusion-layer model was applied in the simulation^39^. The boundary condition at the diffusion layer is considered constant and equilibrated with external environment.

The periodicity *p*, defined as the distance between two neighboring wires, is a variable used for parametric sweep. The length (*l*) and diameter (*d*) of the wire were set to be 50 and 4 µm, respectively, given practical issues of device fabrication (see Supplementary Note 2). The diffusion layer thickness *d*, defined as the distance from any point on the boundary of diffusion layer to the nearest electrode surface, was set to be 20 µm. Such a value is determined based on the experimentally determined O_2_ profile (blue trace in Fig. 2f), which displays a near-linear O_2_ profile within about 20 µm away from the electrode.

A constant electrochemical voltage *E*_appl_ was applied on the electrode’s surface. The reaction of electrochemical reduction at the electrode’s surface proceeds as

$${\mathrm{O}}_2{\mathrm{ + 4}}\,{\mathrm{H}}^ + {\mathrm{ + 4e}}^ - \to {\mathrm{2H}}_2{\mathrm{O}}$$

Our model was constructed based on the following equations:

Governing differential equations:

1 $$\frac{{\partial [{\mathrm{O}}_2]}}{{\partial t}} = D_{{\mathrm{O}}_2}\nabla ^2\left[ {{\mathrm{O}}_2} \right] = 0$$

Boundary conditions

At the electrode: (concentration-dependent Tafel equation)

2 $$i_{{\mathrm{{loc}}}} = - i_0\frac{{\left[ {{\mathrm{O}}_2} \right]}}{{c_{{\mathrm{{O}}}_2}}}{\mathrm{{exp}}}\left( {\frac{{ - \alpha _{\mathrm{{c}}}F\eta }}{{{\mathrm{{RT}}}}}} \right)\,{\mathrm{and}}\,J_{{\mathrm{{O}}}_2} = \frac{{i_{{\mathrm{{loc}}}}}}{{4F}}$$

At the boundary of diffusion layer

3 $$\left[ {{\mathrm{O}}_2} \right] = c_{{\mathrm{O}}_2}$$

At the other boundaries of the periodic unit cell:

Periodic boundary conditions

where *i*_loc_ is the local current density of oxygen reduction reaction at a given location on the electrode boundary, $J_{{\mathrm{O}}_{2}}$, the flux of O_2_ mass transport at a given location on the electrode boundary, *i*_0_ = 3.25 × 10^─7^ mA cm^─2^, the exchange current density, determined by Tafel analysis of electrochemical measurement of Pt-sputtered wire array electrode in bulk medium (Supplementary Note 9 and Supplementary Fig. 12), *α*_c_ = 0.5 the transfer coefficient, *F* is the Faraday constant, *R* is the gas constant, *T* is the temperature, [O_2_] the local O_2_ concentration, and $c_{\mathrm{{{O}}}_{2}}$ the solubility of O_2_ in water when equilibrated with air. The over potential *η*, is the difference between *E*_appl_ and the standard redox potential of O_2_/H_2_O, $E_{{\mathrm{{O}}}_{2}/{\mathrm{H}}_2 {\mathrm{O}}}^0$. Due to the high buffer capability of the medium compared to the demand of proton for O_2_ reduction, the proton concentration is considered constant throughout the system (Supplementary Note 10). Therefore, the proton concentration in the system is assumed to be constant in the simulation. Additional discussion on the choice of the model can be found in Supplementary Note 11. The values of parameters used for simulation are listed in Supplementary Table 2.

### Preparation procedure of microwire array electrode

Si wire array was prepared using photolithography followed by reactive ion etching^28^. 500 μm-thick, 4′ Si wafer (p-type, boron-doped, 〈100〉 facet-oriented, 1–10 Ω cm^–1^, University wafer) was patterned by a contact aligner (Carl Suss MA6) with photoresist (MicroChemicals AZ5214E), and was then developed with MicroChemicals AZ 400K. The microwire array morphology was created by a subsequent reactive ion etching (Unaxis Versaline, FDSE III). A layer of protective silicon dioxide layer on the surface of wire array was created by thermal annealing in air at 1050 °C for 9 h in a tube furnace (Lindberg/Blue M, Fisher Scientific). A conformal layer of ITO with 200-nm thickness was coated via reactive sputtering using ULVAC RF sputtering system. Pt particles of 2 nm thickness was deposited on the wire’s surface with a Anatech Hummer 6.2 sputtering system. The morphology of prepared wire array was characterized by a SEM (JEOL JSM-6700F) equipped with energy dispersive X-ray spectroscopy (EDS, Ametek).

### Electrochemical measurement in bulk solution

LSV of the wire-array (Supplementary Fig. 3) in bulk minimal medium was obtained using a typical three-electrode set-up on Gamry 1010B potentiostat system. The wire array was used as working electrode, a piece of silver paint-coated ITO glass was used as reference electrode, same as the pseudo-reference electrode used in the electrochemical chamber. Pt wire was used as counter electrode. Air/argon-saturated minimal medium was prepared by bubbling air/argon in minimal medium for 30 min. Such minimal medium was used as electrolyte. The LSV was obtained by scanning from 1.0 to −0.5 V vs. RHE at the speed of 5 mV s^─1^.

### Construction of electrochemical setup on confocal microscope

An electrochemical setup under a confocal microscope was constructed for both the experimental validation of O_2_ gradient and the characterization of electricity-driven N_2_ fixation (Supplementary Fig. 4). A three-electrode electrochemical configuration was fitted into a flow chamber with a chamber thickness of 200 µm (Fig. 2b and Supplementary Fig. 4). The wire array electrode constitutes the working electrode. An ITO-coated glass coverslip (#1.5, 40 mm × 22 mm, SPI supplies), wet etched with 6 M HCl into two isolated electrodes, was used as the top cover of the chamber. One section of the ITO was sputtered with 7-nm Pt and used as the counter electrode; the other one, coated with silver paint (Ted Pella, Inc) at the downstream direction, was designated as the Ag pseudo-reference electrode. Prior to experiment, the Ag pseudo-reference electrode was calibrated in the ZoBell’s solution (see method below). The pseudo-references maintain a stable 0.31–0.32 V vs. standard hydrogen electrode (SHE) throughout the 120 h of electrochemical operation. A syringe or peristaltic pump was employed to feed the liquid medium into the chamber at a fixed flow rate. A Gamry Interface 1010B was applied for electrochemical characterization. The whole setup is optically transparent and mounted to an inverted laser confocal microscope (Leica SP8 SMD), allowing the use of objectives with working distance of 680 µm.

### Characterization of the Ag pseudo-reference

To characterize the electrode potential vs. SHE, and test the stability of pseudo-reference electrodes, prepared by Ag paste, cyclic voltammetry was conducted in a standard ZoBell’s solution^40^ using our Ag pseudo-reference before and after a 120-h operation as reference electrode. In each experiment of cyclic voltammetry, the device is exposed to ZoBell’s solution, which is composed of 3.3 mM K_3_Fe(CN)_6_, 3.3 mM K_4_Fe(CN)_6_, and 0.1 M KCl^41^. Cyclic voltammogram was recorded at a scan rate of 20 mV s^−1^ using the pseudo-reference electrode as the reference, a 3.2 mm-diameter glassy carbon electrode as working electrode, and a Pt wire as the counter electrode (Supplementary Fig. 13). A minimal amount of time for the exposure of ZoBell’s solution was ensured so that the exposure per se will not alter the redox potential of the pseudo-reference electrodes. As the ZoBell’s solution is known to have a redox potential of +0.43 V vs. SHE^41^, the potentials of pseudo-reference electrode were able to be obtained by determining the mid-point potential of Fe(III)/Fe(II) redox couple in the cyclic voltammograms. Representative cyclic voltammograms before (Supplementary Fig. 13, black line, mid-point potential = 113 mV vs. reference) and after (Supplementary Fig. 13, red line, mid-point potential = 117 mV vs. reference) 120-h operation display minimal differences in the mid-point potential of Fe(III)/Fe(II) redox couple, illustrating that the pseudo-reference electrodes maintain a stable 0.31–0.32 V vs. SHE over an extended period of time.

### Experimental validation of O_2_ gradients

In situ phosphorescence and lifetime imaging of Ru(phen)_3_Cl_2_ was employed in the microwire electrode to validate the proposed O_2_ gradients (Fig. 2b). The phosphorescence was monitored at 570–650 nm by a 470-nm laser excitation, with a ×20 immersion type objective lens (Leica ×20 HC PL APO IMM CS2 NA/0.75). 0.1 mM Ru(phen)_3_Cl_2_ (Sigma-Aldrich) in 1× phosphate buffered saline (PBS, pH = 7.0, Supplementary Table 3) was being flowed through the chamber with a syringe pump (New Era Pump Systems, Inc.) at a flow rate of 2 mL min^–1^.

The intensity of phosphorescence emission was collected by photon multiplier tube detectors (PMT). The data was acquired using Leica Application Suite X (LASX) on *x–z–t* mode at a spatial resolution of 146 nm/pixel, taking *x–z* cross-sectional images at the speed of 2.773 s per frame (Fig. 2c, d). The absolute phosphorescent intensity is lower at the region near the bottom of the wire array as shown in Fig. 2c, d, which indicate the intensity *I*_em_ fluorescent emission is prone to possible interference from the optical absorption of Si wire array, as the Si wire array can absorb some of the incident and emitted photons. Therefore, the quantitative analysis of local O_2_ concentration was conducted by PLIM, in which the lifetime measurement is independent of light intensity. The spatially resolved lifetime measured was conducted using time-correlated single photon counting module (HydraHarp, PicoQuant) integrated to the microscope. A 470-nm pulsed laser was used as excitation light and the emitted photon between 570 and 650 nm were collected using Leica hybrid detector (HyD) coupled with the TCSPC module. The spatial lifetime information was processed by the fluorescent lifetime imaging software Symphotime (PicoQuant). Single exponential tail fitting was used to analyze phosphorescent decay and calculate lifetime. Our measurements were calibrated against standard solutions of known O_2_ concentration without applied electric potential. A linear fit between the reciprocal lifetime *τ*^–1^ against [O_2_] (Supplementary Fig. 6) suggests high fidelity of the method to quantify O_2_ concentrations. Due to the laminar flow pattern within the flow cell, the generated O_2_ near the counter electrode (Supplementary Fig. 14) does not affect the O_2_ gradient near the wire array (Supplementary Note 12). Results measured in PBS is representative for results measured in microbial medium used for N_2_ fixation experiments, as the quantification method is proven not sensitive to the composition of medium (Supplementary Note 13 and Supplementary Fig. 15). Detailed technical parameters for imaging process can be found in Supplementary Methods.

### Bacterial strains and culturing methods

*X. autotrophicus* (*X. autotrophicus*, ATCC 35674) was purchased from American Type Culture Collection (ATCC), *B. japonicum* (USDA DES 122, DSM 1755) was purchased from German Collection of Microorganisms and Cell Cultures (DSMZ), and *B. japonicum*-*H1* (*B. japonicum-H1*)^34^ was courtesy of Prof. Ann Hirsch. Microbes were cultured using the following procedure^5,13^. Bacterial samples were thawed from frozen stock (Supplementary Methods) and incubated aerobically in succinate nutrient broth (Supplementary Table 4) at 30 °C for overnight, reaching an optical density of 600 nm (OD_600_) higher than 1. The cultured cells were harvested by centrifugation and re-suspended at an initial OD_600_ = 0.25 in a pH = 7.0 liquid medium deprived of any nitrogen and organic carbon sources, whose recipes are available in Supplementary Tables 5 and 6 for *X. autotrophicus* and *B. japonicum*, respectively. The cultures were incubated under a hypoxic condition (3% O_2_, 20% H_2_, 17% CO_2_, 60% N_2_) at 30 °C for 6 days in a sealed jar (Vacu-Quik Jar, Almore). The resultant OD_600_ after 6 days were about 0.7–1.0. As the *B. japonicum-H1* strain is incapable to fix N_2_, the microbial growth medium was supplemented with (NH_4_)_2_SO_4_ at a concentration of 1.25 g L^−1^ and cultured for 6 days in a gas environment with 10% O_2_, 40% H_2_, 10% CO_2_, and 40% N_2_.

### Electricity-powered biological|inorganic N_2_ fixation

Diazotroph cultures (OD_600_ = 1.0), starved at room temperature for 6 h, were introduced into the electrochemical chamber equipped with microwire array, which was sterilized by isopropyl alcohol before use. The microbes were allowed to associate with the wire array for one hour, before the chamber were rinsed with cell-free medium to remove excessive planktonic cells. The association was probably due to the adhesion between microbes and wire array (Supplementary Note 14). The medium utilized here was pre-equilibrated with air before injection and deprived of any nitrogen and organic carbon sources, so that any observable biomass accumulation is a result of N_2_ and CO_2_ fixation. After the inoculation procedure, an electrochemical bias (*E*_appl_) was applied to the electrochemical setup while a culture medium, pre-saturated with air, was flown through the chamber at a flow rate of 2 mL min^−1^. A typical experiment was conducted for at least 120 h at 30 °C. The current densities during the experiments were recorded (Supplementary Fig. 16). Biofilm formation was not found during the period of 120-h incubation (Supplementary Note 15).

The microbial culture within the wire array was characterized by a variety of methods. Fluorescence imaging was employed to monitor the microbial population, by staining the microbes with a 1x PBS solution of 10 μM Rhodamine 6G (Alfa Aesar) solution for 15 min before flushed out. Fluorescence emission at 570–650 nm was collected with the use of a 532-nm laser excitation. The images were taken on *x–y–z* mode, taking *x–y* cross-sectional image on different *z*-axis position at a spatial resolution of 146 nm/pixel with a *z* increment of 1 µm. Such a fluorescence characterization leads to the determination the changes of microbial cell counts (ΔCC) in the wire array. Colorimetric assays that were used in environmental monitoring^42,43^ were employed to yield the changes of total nitrogen content (ΔTN) and the changes of total organic carbon (ΔTOC).

### Estimation of bacterial growth within the wire array

We estimated the number of bacteria within the wire array using phosphorescence image obtained from confocal microscopy. For each *x–y* cross-sectional image, we estimate the total cell number by the total pixel number considered “light”, judged by a certain intensity threshold. The fluorescent image was processed using ImageJ. White noise was removed using “remove outliers” command under the settings of 2.0 pixels radius and 30 as outlier threshold. The image was then transformed into a binary figure with the intensity threshold, in which “light” pixels are “1”s and “dark” pixels are “0”s. The sum of all “light” pixels in an image reveals how many pixels are representing stained bacterial cells. The higher the sum, the more cells are in the image, as displayed in Fig. 3e. To quantify the absolute cell number, we used external standard. Briefly, a “standard image” was captured, using the same method and scan resolution as used in our microscopic observations, on a single-layered bacterial sample with each bacterium separated from each other. The total cell number in the image was manually counted, and the sum of “light” pixels was calculated after the same image-processing method mentioned above. The average pixel number per bacteria (pix/bacteria) was then obtained. For each *x–y* cross-sectional image, the sum of “light” pixels is divided by pix/bacteria to calculate the total number of bacteria in one image. This may lead to slight underestimation of bacterial numbers, due to the assumption that there was no overlap between bacteria in our captured images. To estimate the overall cell number in the three-dimensional space within the wire array, we assume that from the bottom of wire to the top of wire a new batch of bacteria will be observed with every 5 μm increase in *z*, as bacteria that were more than 5 μm away from focus plane will not appear in the cross-sectional image. Thus the overall number within the observed wire array would be *N*_average_ × (*l*/5 μm), where *l* is the length of wire and *N*_average_ is the average bacterial number in *x–y* cross-sectional images acquired at different distances from wire bottom (*z*). As we noticed the bacterial growth at different *z* were not equal (as shown in Fig. 3e–g), the average cell number *N*_average_ was taken by averaging the cell number in *x–y* images at *z* = 0, 10, 20, 30, 40, and 50 μm for a *l* = 50 μm wire array. The cell number in terms of density was acquired by dividing the overall cell number by the volume of imaged space. The increase in cell number was calculated by the difference of cell number after 5 days incubation and the cell number right after inoculation.

### Total nitrogen (TN) and organic carbon determination

The general method to determine TN and total organic carbon (TOC) retained in biomass within wire array is multiplying the TN and TOC of a single bacterium by the number density of bacteria within the wire array, which is determined using method described in the method above. First, TN and TOC of bacterial culture with known cell number are determined by absorbance spectrometry using test kits (Hach, kit #10071 for TN and Hach, kit #10627 for organic carbon). 5-day lithotrophically cultured bacterial cells are harvested from culture using centrifugation and re-suspended in nitrogen and organic carbon-free medium. The OD_600_ of bacteria suspension was adjusted to 0.7 before testing. The measuring procedure is briefly described here. For TN quantification, 2 mL of bacterial suspension is first digested using alkaline persulfate solution. This step quantitatively transforms all nitrogen species into nitride. Then the solution was acidified to so that all nitrides turns to nitric acid. The nitric acid was then reacted with phenol, as this quantitative reaction yields yellow colored benzoquinone. The absorbance of generated benzoquinone at 410 nm is thus dependent on the concentration of nitric acid, which relates to the TN amount in bacteria. A calibration curve correlating the absorbance at 410 nm and TN concentration (Supplementary Fig. 17) was constructed using the kit on different standard nitrogen sample solutions (ammonium *p*-toluenesulfonate, Hach). For TOC quantification, 2 mL of bacterial suspension was first acidified and heated to remove inorganic carbonates. Then the solution was digested using persulfate to oxidize all organic carbon into carbon dioxide. The generated carbon dioxide was quantitatively absorbed using acid–base indicator solution. The carbonic acid originated from absorbed carbon dioxide will then react with the indicator and yield a product (the conjugated acid of the indicator) that show increase in the absorbance at 435 nm. A calibration curve correlating the absorbance at 435 nm and TOC concentration (Supplementary Fig. 18) was constructed using the kit on different standard nitrogen sample solutions (TOC standard, 1000 mg L^–1^, Hach). The absorbance was measured using a Hewlett-Packard 8453 UV–vis spectrometer.

Second, the number density of bacteria per OD_600_ were 2.8 × 10^8^ mL^─1^ for *X. autotrophicus*^12^, and 1.0 × 10^9^ mL^─1^ for *B. japonicum*. The number of *B. japonicum* per OD_600_ was determined using flow cytometry, which was run on a BD LSRII (IMED) analytic flow cytometer. The values of nitrogen content of each single cell were calculated using the overall TN or TOC of a culture with certain OD_600_ divided by the number of bacterium.

The TN and organic carbon for single bacterium cell (*n* = 3):

**Table:** 

Strain	Total nitrogen (mg cell^─1^)	Total organic carbon (mg cell^─1^)
*X. autotrophicus*	1.5 ± 0.0 × 10^─10^	6.4 ± 0.3 × 10^─10^
*B. japonicum*	4.4 ± 0.3 × 10^─11^	1.6 ± 0.0 × 10^─10^

In order to validate the quantification method described above, when necessary, the microfluidic setup was carefully dissembled. The microorganisms in the setup were carefully extracted by rinsing with minimal medium and mechanically scratching microwire array electrodes. As the microwire array electrodes were made of Si, SiO_2_, indium tin oxide (ITO), and Pt, the mechanically detached particles of wire array will not interfere with the oxidative procedure for the measurement of TN in the microorganisms. Similar methods as mentioned above was applied on the detached microbes. The overall microbial nitrogen, after 120 h under *E*_apple_ = −0.15 V vs. RHE in *p* = 15 μm microwire array, were 2.48 ± 0.52 and 0.503 ± 0.077 μg for *X. autotrophicus* and *B. japonicum*, respectively (*n* = 2), which corresponds to 992 and 201 mg L^─1^ within the wire array for *X. autotrophicus* and *B. japonicum*, respectively. These values are very close to the values measured using the general method mentioned above (975 and 196 mg L^−1^ N for *X. autotrophicus* and *B. japonicum*, respectively), validating our method of nitrogen quantification mentioned above.

The amount of free ammonia in the effluent of our N_2_ fixation system is also determined by absorbance spectrometry using test kit (Hach, TNTplus 830). The flow cell with *p* = 15 μm microwire array is inoculated with bacteria and run under *E*_apple_ = −0.15 V vs. RHE for 120 h, same as described in methods. The flow speed was 2 mL min^─1^. A total of 20 mL of medium was used and the effluent was circulated in the system to accumulate released ammonia to a detectable level. The increase of ammonia nitrogen was determined by absorbance spectrometry (Hach, Method 10205). 5 mL of effluent was added into the reaction mixture of the kit. After thoroughly mixing, the mixture was reacted for 15 min. The final solution’s absorbance at 694 nm was used to determine the amount of ammonia. The increase of ammonia nitrogen was found to be 0.017 ± 0.003 mg L^−1^ in the effluent of *B. japonicum* (*n* = 2). In the effluent of *X. autotrophicus*, we did not find detectable amount of ammonia. Therefore, it is considered as 0 mg L^−1^ in our calculations for activities and efficiencies. Comparing with the value of 187 mg L^−1^ (*B. japonicum*) for the increased biomass nitrogen under the same condition, the free ammonia accounts for more than 40% of the TN fixed by *B. japonicum*.

### N_2_ fixation activity calculation

The activity of N_2_ fixation displayed in Supplementary Table 1 is calculated in two units: mg N_2_ per liter per hour (*rN*_V_) and mg N_2_ per g dry weight biomass per hour (*rN*_m_). The experimental results for microbes cultured using *p* = 15 μm microwire array under *E*_apple_ = −0.15 V vs. RHE for 120 h (Fig. 3d, entries 1 and 2) are used.

*rN*_V_ was calculated by the volumetric increase of total nitrogen (ΔTN) in biomass and free ammonia released in medium:

4 $$rN_{\mathrm{{V}}} = \frac{{\Delta {\mathrm{TN}}_{{\mathrm{bio}}} \times V_{{\mathrm{wire}}\,{\mathrm{array}}} + \Delta {\mathrm{TN}}_{{\mathrm{medium}}} \times V_{{\mathrm{medium}}}}}{{V_{{\mathrm{wire}}\,{\mathrm{array}}} \times t}}$$

where ΔTN_bio_ is the increase of TN in microbe cells within the array (5.7 × 10^2^ and 1.9 × 10^2^ mg L^─1^ for *X. autotrophicus* and *B. japonicum*, respectively), *V*_wire array_ the volume of wire array (2.5 × 10^─6^ L), ΔTN_medium_ the increase of TN as ammonia in microbial growth medium (0 mg L^−1^ for *X. autotrophicus* and 0.017 mg L^−1^ for *B. japonicum*), *V*_medium_ the volume of medium circulated in the device during operation (20 mL), *t* the overall incubation time (120 h). From the equation we calculate the *rN*_*V*_ values, which are 4.8 and 2.7 mg L^─1^ h^─1^ for *X. autotrophicus* and *B. japonicum*, respectively.

*rN*_m_ was calculated using the sum of ΔTN and free nitrogen in effluent divided by the bacterial average dry mass (TN_average_/15%):

5 $$rN_{\mathrm{{m}}} = \frac{{\Delta {\mathrm{TN}}_{{\mathrm{bio}}} \times V_{{\mathrm{wire}}\,{\mathrm{array}}} + \Delta {\mathrm{TN}}_{{\mathrm{medium}}} \times V_{{\mathrm{medium}}}}}{{{\mathrm{TN}}_{{\mathrm{average}}} \times V_{{\mathrm{wire}}\,{\mathrm{array}}} \times t/15\% }} \times 1000$$

under the assumption that nitrogen constitutes 15% of bacterial dry mass. TN_average_ is the average nitrogen content in microbes over the growth time. We assume exponential growth and take the average of the biomass amount throughout the 120-h incubation as the TN_average_ (644 and 61 mg L^−1^ for *X. autotrophicus* and *B. japonicum*, respectively). ΔTN_bio_ is the increase of TN in microbe cells within the array (5.7 × 10^2^ and 1.9 × 10^2^ mg L^─1^ for *X. autotrophicus* and *B. japonicum*, respectively), *V*_wire array_ the volume of wire array (2.5 × 10^─6^ L), ΔTN_medium_ the increase of TN as ammonia in microbial growth medium (0 mg L^−1^ for *X. autotrophicus* and 0.017 mg L^−1^ for *B. japonicum*), *V*_medium_ the volume of medium circulated in the device during operation (20 mL), *t* the overall incubation time (120 h). The values of TN of inoculum. The *rN*_m_ values are 1.1 and 6.5 mg g^─1^ h^─1^ for *X. autotrophicus* and *B. japonicum*, respectively.

### Calculation of Faradaic efficiency (FE) and energy cost

We define FE as the percentage of electron that has been used to reduce N_2_ and CO_2_ to form microbial biomass and secreted ammonia. The FE is calculated as follows:

6 $${\mathrm{FE}} = \frac{{F\left[ {3\frac{{\Delta {\mathrm{TN}}_{{\mathrm{bio}}} \times V_{{\mathrm{wire}}\,{\mathrm{array}}} + \Delta {\mathrm{TN}}_{{\mathrm{medium}}} \times V_{{\mathrm{medium}}}}}{{M_{\mathrm{N}}}} + 4\frac{{\Delta {\mathrm{TOC}}_{{\mathrm{bio}}} \times V_{{\mathrm{wire}}\,{\mathrm{array}}}}}{{M_{\mathrm{C}}}}} \right]}}{{i \times A \times t}} \times 100\%$$

where ΔTN_bio_ is the increase of TN in microbe cells within the array (5.7 × 10^2^ and 1.9 × 10^2^ mg L^─1^ for *X. autotrophicus* and *B. japonicum*, respectively), *V*_wire array_ the volume of wire array (2.5 × 10^─6^ L), ΔTN_medium_ the increase of TN as ammonia in microbial growth medium (0 mg L^−1^ for *X. autotrophicus* and 0.017 mg L^−1^ for *B. japonicum*), *V*_medium_ the volume of medium circulated in the device during operation (20 mL), ΔTOC_bio_ the increase of TOC in microbe cells within the array (2.5 × 10^3^ and 6.9 × 10^2^ mg L^−1^ for *X. autotrophicus* and *B. japonicum*, respectively), *i*, the overall current density that pass through the wire array electrode during the 120 h operation of our hybrid system (ca. 80 μA cm^−2^), *A*, the projected area of wire array electrode (0.5 cm^2^), *t* the overall operation time (120 h, correspondingly 432,000 s), *F*, Faraday constant (96,485 C mol^−1^), *M*_N_ and *M*_C_, the molar atomic weight of N (14 g mol^−1^) and C (12 g mol^−1^), respectively. The FEs are calculated to be 1.4% and 0.42% for *X. autotrophicus* and *B. japonicum*, respectively (*p* = 15 µm; *E*_appl_ = −0.15 V vs. RHE).

The energy cost of nitrogen fixation in terms of kJ/g nitrogen is estimated using the following equation:

7 $$E_{\mathrm{{N}}} = \frac{{i \times t \times A \times U}}{{1000 \times \left( {\Delta {\mathrm{TN}}_{{\mathrm{bio}}} \times V_{{\mathrm{wire}}\,{\mathrm{array}}} + \Delta {\mathrm{TN}}_{{\mathrm{medium}}} \times V_{{\mathrm{medium}}}} \right)}}$$

where *E*_N_ is the energy cost (kJ/g nitrogen), *i* the current density (ca. 80 μA cm^−2^), *A*, the projected area of wire array electrode (0.5 cm^2^), *t* the total incubation time with applied voltage (120 h, correspondingly 432,000 s), *U* the applied potential between the working and the counter electrodes, here we assume *U* = 1.23 V for water splitting, ΔTN_bio_ is the increase of TN in microbe cells within the array (5.7 × 10^2^ and 1.9 × 10^2^ mg L^─1^ for *X. autotrophicus* and *B. japonicum*, respectively), *V*_wire array_ the volume of wire array (2.5 × 10^─6^ L), ΔTN_medium_ the increase of TN as ammonia in microbial growth medium (0 mg L^−1^ for *X. autotrophicus* and 0.017 mg L^−1^ for *B. japonicum*), *V*_medium_ the volume of culture medium circulated in the device during operation (20 mL). Here we consider the nitrogen contents retained in biomass and secreted into the circulating medium. Following the procedure described above, the overall energy cost is 1.5 × 10^4^ and 2.6 × 10^4^ kJ/g nitrogen for *X. autotrophicus* and *B. japonicum* strains, respectively (*p* = 15 µm; *E*_appl_ = −0.15 V vs. RHE).

## Supplementary information

Supplementary Information

## Data Availability

The datasets generated or analyzed during the current study are available from the corresponding author upon reasonable request. The source data underlying Figs. 1d, 2e, 2f, 3d, and 3e, and Supplementary Figs. 2, 3, 6, 9, 10, 12, 13, 15–18 are provided as Source Data file, which can be found following the url: 10.6084/m9.figshare.11879715.v2.

## Code availability

The code used in the current study are available from the corresponding author upon reasonable request.
