# Supplementary material for: Electricity-powered artificial root nodule
Source: Nat Commun. 2020 Mar 20;11:1505. doi: 10.1038/s41467-020-15314-9 (PMC7083970; doi:10.1038/s41467-020-15314-9)
Supplement: Supplementary file 1 — Supplementary Information [file 41467_2020_15314_MOESM1_ESM.pdf]

Supplementary Information

Electricity-Powered Artificial Root Nodule

Lu, *et al.*

Table of contents

Content	Page
1. Supplementary Methods	S4
1.1. Equipment and settings for microscopic imaging	S4
1.2. Preparation of microbial frozen stock	S4
2. Supplementary Notes	S6
2.1. Supplementary Note 1	S6
2.2. Supplementary Note 2	S6
2.3. Supplementary Note 3	S7
2.4. Supplementary Note 4	S7
2.5. Supplementary Note 5	S8
2.6. Supplementary Note 6	S8
2.7. Supplementary Note 7	S9
2.8. Supplementary Note 8	S9
2.9. Supplementary Note 9	S9
2.10. Supplementary Note 10	S9
2.11. Supplementary Note 11	S10
2.12. Supplementary Note 12	S12
2.13. Supplementary Note 13	S12
2.14. Supplementary Note 14	S13
2.15. Supplementary Note 15	S13
3. Supplementary tables	S14
3.1. Supplementary Table 1	S14
3.2. Supplementary Table 2	S15
3.3. Supplementary Table 3	S16
3.4. Supplementary Table 4	S17
3.5. Supplementary Table 5	S18
3.6. Supplementary Table 6	S19
3.7. Supplementary Table 7	S20
3.8. Supplementary Table 8	S21
4. Supplementary figures	S22
4.1. Supplementary Fig. 1	S22
4.2. Supplementary Fig. 2	S23

4.3. Supplementary Fig. 3 S24
4.4. Supplementary Fig. 4 S25
4.5. Supplementary Fig. 5 S26
4.6. Supplementary Fig. 6 S27
4.7. Supplementary Fig. 7 S28
4.8. Supplementary Fig. 8 S29
4.9. Supplementary Fig. 9 S30
4.10. Supplementary Fig. 10 S31
4.11. Supplementary Fig. 11 S32
4.12. Supplementary Fig. 12 S33
4.13. Supplementary Fig. 13 S34
4.14. Supplementary Fig. 14 S35
4.15. Supplementary Fig. 15 S36
4.16. Supplementary Fig. 16 S37
4.17. Supplementary Fig. 17 S38
4.18. Supplementary Fig. 18 S39
Reference S40

1. Supplementary methods

1.1. Equipment and settings for imaging

In general, a Leica SP8 SMT model confocal scanning laser microscope was used for imaging purpose for this work. The light source was a white-light laser capable to emit 470 – 670 nm (SuperK EXTREME EXW-12, NKT Photonics). The wave length selection for excitation and emission was fulfilled by the acousto-optic modulators (AOM) built in the microscope.

Phosphorescence images shown in Fig. 2c, 2d and Supplementary Fig. 5 were captured using Leica Application Suits X (LASX) software. 470 nm laser was used as the excitation light and emissions from 570 nm ~ 650 nm were collected into photon multiplier tubes (PMTs). The scanner was running on *x*-*z*-*t* mode with spatial resolution of 146 nm per pixel and temporal resolution of 2.773 s per frame. The resulted image was 256-bit grey scale mapping of intensity. The image was processed using imageJ. The contrast was adjusted linearly. The photo was then pseudo-coloured blue using Adobe Photoshop. The processed image was cropped to 80 × 80 μm^2 before used. The fluorophore used was $Ru(phen)_3^{2+}$.

The images showing in Fig. 3b, 3c, 3f, 3g, and Supplementary Fig. 7 and 8 were captured using Leica Application Suits X (LASX) software. 532 nm laser was used as the excitation light and emissions from 570 nm ~ 650 nm were collected into photon multiplier tubes (PMTs). The scanner was running on *x*-*y*-*z* mode with *x*-*y* spatial resolution of 146 nm per pixel and *z* resolution of 1 μm per frame in the *z*-stack. The resulted image was 256-bit grey scale mapping of intensity. The image was processed using imageJ. The contrast was adjusted linearly. White noise was removed using “remove outliers” command under the settings of 2.0 pixels radius and 30 as threshold. The processed image was pseudo-coloured green using Adobe Illustrator and directly used without cropping, showing the full 150 × 150 μm^2 field of view. The fluorophore used was Rhodamine 6G.

1.2. Preparation of microbial frozen stock

Xanthobacter autotrophicus (*X. autotrophicus*, ATCC 35674) was obtained from American Type Culture Collection (ATCC). *Bradyrhizobium japonicum* DES 122 (*B. japonicum*, DSM 1755) was obtained from German Collection of Microorganisms and Cell Cultures (DSMZ). *Bradyrhizobium japonicum-nifH* was donated by Prof. Ann Hirsch. Both samples were received as freeze dried powder. The reviving process was based on instructions from the provider. The freeze dried powder were first suspended in succinate nutrient broth (Supplementary Table 4) and incubated overnight in 30 ℃ to reach steady growth phase. The culture was then inoculated on to succinate agar plates (1.5% agar in succinate nutrient broth)

using an inoculation loop and further incubated for 2 to 3 days until significant yellow (for *X. autotrophicus*) or pale yellow (for *B. japonicum*) colonies could be observed. A single colony is picked and suspended in succinate nutrient broth and incubated under 30 ℃ for overnight. The culture was then mixed with glycerol ($V_{culture}$: $V_{glucerol}$ = 4 : 1) before frozen in −80 ℃ as the frozen stock. Bacteria used in experiments were prepared from frozen stock as described in Methods in main text.

2. Supplementary Notes

2.1. Supplementary Note 1

The advantage of using microwire array system compared to a biofilm system.

Besides our microwire array system, another approach that utilizes biofilm to fix nitrogen has been reported recently.[1-4] The formation of biofilm is usually concurrent with the introduction of other non-N_2-fixing microbial strains[1,4], the synthesis of extracellular polymeric substances,[3,5] as well as changes in genetic regulation of N_2 fixation.[6] These factors probably contribute to our observation that the rate of nitrogen fixation reported in existing literature using electrochemical method (2.6 $mg \cdot L^{-1} \cdot hr^{-1}$ and 0.008 $mg \cdot L^{-1} \cdot hr^{-1}$ in reference 11 and 12) are smaller than the value reported in our current work (4.8 $mg \cdot L^{-1} \cdot hr^{-1}$ for *X. autotrophicus* strain, see Methods). Moreover, as fundamentally both biofilm and our approach require a material's surface for attachment, we consider the scalability of these two approaches comparable. In the future, scaling up our integrated approach can be much accelerated by taking advantages of the know-how developed in biofilm catalysis.[5]

Our current approach offers a more general method of employing electricity to culture N_2-fixing diazotrophs, as compared to the alternative approach based on biofilms. The biofilm-based approach requires that the diazotroph maintains its N_2-fixing functionality in the biofilm. Due to the complexity of the genetic regulation of N_2 fixation,[6] not all diazotrophs can remain functional in this approach and to our knowledge up to now only a few examples of biofilm-based N_2-fixation in air are reported.[1,3,4] In our approach, the introduction of inorganic wire electrodes removes the restriction of biofilm formation and allows a broader range of microbes to be incorporated. This is particularly attractive in the context that many diazotrophs as plant growth-promoting microbes[7] are desirable not only because they fix N_2 but also because they secrete beneficial plant hormones. We posit that our approach can be a general method for microbes that deliver nitrogen fertilizer and/or plant hormones, which will not be genetically expressed with the formation of biofilm.

2.2. Supplementary Note 2

Design rationale of the wire array morphology

Here we aim to discuss the rationale underlying our design of the wire array morphology. Three major factors are taken into the consideration: 1) the efficacy of constructing O_2 gradient without significantly hampering the mass transport of N_2; 2) the practicality of device characterization; 3) the practicality of wire array synthesis. First, a wire array of a suitable wire length l is desired, as a short wire is not effective to create the hypoxic domain for biological N_2 fixation while an excessively long wire will mitigate N_2 transport. As the diffusion layer

thickness is about 20 μm in our setup, we consider that wires of l = 50 μm are suitable as a proof-of-concept. Second, the validation of O_2 gradient and the characterization of microbial population relies on optical microscopes, which demands a sufficiently high transparency of the device. A wire array of l = 50 μm with periodicity p larger than 10 μm are needed in order to satisfy this requirement, as longer wires with smaller periodicity pose additional characterization challenges. Third, as a proof-of-concept, chemical etching was used to create the desirable microstructures with high fidelity. Such an etching method will difficult to yield long wires of smaller diameters. Due to this consideration, a diameter d = 4 μm was set to facilitate sample preparation while leave enough "open space" among the wire arrays for the diazotrophic microbial population. In general, the concept of electricity-driven artificial root-nodule is not limited by these practical constraints. We envision that advanced preparation techniques are capable to scale up the device with lower cost, which will be beneficial to the proposed application in the long run.

2.3. Supplementary Note 3

Comparison of doubling time between bacteria grown in the artificial root nodule and grown in autotrophic environment.

The literature reported doubling time for planktonic *B. japonicum* and *X. autotrophicus* are 10 and 12 hours, respectively, in liquid medium under autotrophic conditions.[8,9] Our experimental observation leads to doubling times of about 28 and 90 hr, respectively. While our observed doubling times are larger than the literature values, we would like to note that these literature values were obtained under optimal environments with well-defined conditions (*e.g.* relatively low cell density and strictly controlled atmosphere). In contrast, the heterogeneity of our environment in air may yield additional oxidative stress for the microbial growth.

Nonetheless, the slowed microbial growth in comparison to aqueous cultures in literature is not in conflict with the observed rate of N_2 fixation, higher than the ones in symbiotic root nodules, as noted in Supplementary Table 1. As speculated in literature,[10-12] it is likely that the absence of plant tissue and their regulation on microbial metabolism in the natural symbiotic system helps to maintain the high rate of N_2 fixation observed in our system.

2.4. Supplementary Note 4

Comparison between energy efficiencies of electricity-powered artificial root nodule and natural root nodule

Though the energy cost in our system is seemingly large (1.5×10^4 and 2.6×10^4 kJ per g nitrogen for *X. autotrophicus* and *B. japonicum* strains, respectively), our biological | inorganic hybrid systems are already much more efficient than the symbiotic systems in natural root nodules.

The energy costs of our approach are lower than the value for the natural symbiotic N_2 fixation (4.2×10^4 ~ 8.4×10^4 kJ per g nitrogen, see the estimation below).

In nature, symbiotic bacteria inside the root nodule consume carbohydrates produced by plant photosynthesis to generate energy to power nitrogen fixation process. The energy efficiency for nitrogen fixation in natural root nodules are based on the following assumptions: 1) The energy efficiency for natural photosynthesis typically do not exceed 1%,[13] and 2) in root nodules, 10 ~20 g of fixed is consumed to power the fixation of 1 g of nitrogen.[14] We take glucose ($C_6H_{12}O_6$), as the representative molecule of carbohydrates. The energy needed to generate one mole of glucose from CO_2 and H_2O, the materials for photosynthesis, is ~3×10^3 kJ.[15] According to assumption 1, the energy needed to produce one mole of glucose is 3×10^5 kJ via plant photosynthesis. A mole of glucose, consumed by the root nodule by respiration, is enough for the fixation of 3.6 ~ 7.2 g nitrogen, according to assumption 2. Based on the above calculation, ignoring the energy lost in the transporting of molecules, 3×10^5 kJ energy is needed for the fixation of 3.6 ~ 7.2 g nitrogen, which corresponds to an energy efficiency of 4.2×10^4 ~ 8.4×10^4 kJ per g nitrogen for the root nodule.

2.5. Supplementary Note 5

Delivery of reducing equivalent to microbes by Pt

Pt is a good electrocatalyst for the reduction of both proton and dioxygen. However, we note that there are literature precedence suggesting that the presence of metals, including Pt, indeed promote the electron transfer between the microbe and inorganic electrode, when both moieties are in close proximity.[16-19] Indeed, the *in situ* fluorescent image in Fig. 3c suggests that after a 120-hr operation the N_2-fixing microbes prefer an electrode/microbe interface. There are many possible explanations for the observation in Fig. 3c, and one of them is the promoted charge transfer due to the presence of Pt. Therefore, we are unable to experimentally exclude the possibility that Pt can facilitate a direct pathway of charge transfer between microbes and electrodes.

2.6. Supplementary Note 6

O_2 gradient under high overpotential.

Although applied electrochemical potential (E_{appl}) does influence the O_2 gradient at low overpotential (Supplementary Fig. 5), when the value of E_{appl} grants large enough overpotential, to some extent the O_2 gradient are no longer sensitive to E_{appl}. This is because that the overall O_2 gradient in the liquid within the wire array is governed by the mass transport of O_2, and follows the theory of porous electrode.[20] The observed O_2 gradients under E_{appl} = 0.5 V *vs.*

Reversible Hydrogen Electrode (RHE) and E_{appl} = −0.15 V *vs.* RHE do not have significant differences (Supplementary Fig. 10).

2.7. Supplementary Note 7

Definition of "porous electrode"

In general, any electrodes that has an intercalating pores should be considered as porous electrodes. This definition includes the majority of nanomaterial-based electrochemical catalysts.[21-27] For example, there are many options of loading catalytic materials on a porous electrode made of graphite granules, and loading Pt nanoparticles on graphite granules remain an industrial practice for electrochemical hydrogen evolution reaction (HER) and oxygen reduction reaction (ORR);[28]. Indeed, most of nanomaterial-based catalysts benefit from the introduction of pores within the electrode, either intentionally or unintentionally, and take advantage of the increased surface area introduced by the pores.

2.8. Supplementary Note 8

Cheaper replacement for Pt as electrochemical catalyst.

Other earth-abundant materials can be used in lieu of Pt in our proposed approach. The design of our platform requires the surface of microwire array electrodes to be electrochemically active towards ORR and HER, while these two catalytic functionalities can be fulfilled by two different types of materials deposited onto the surface of wire electrodes. Under such a guiding principle, many earth-abundant, biocompatible ORR and HER catalysts, such as the cobalt-phosphorous alloy (CoP) for HER and cobalt sulfide (CoS) for ORR, are suitable candidates.[29-35] Incorporating earth-abundant catalysts on microwires will help to scale up the developed platform in the future.

2.9. Supplementary Note 9

Determination of exchange current density (i_0) for ORR

Tafel analysis[36] was implemented to determine the exchange current density i_0 shown in Supplementary Table 2. Tafel analysis was conducted based on the data of linear scan voltammograms (LSV) for electrochemical O_2 reduction with the fabricated wire array electrode (Supplementary Fig. 3). Here we focused on the rising edge of the cathodic current (between 0.65 ~ 0.75 V *vs.* RHE, *i.e.* $|\eta|$ = 0.48 ~ 0.58 V), a practice that mitigates possible interference from the mass transport of O_2. As shown in Supplementary Fig. 12 (black dots), Tafel analysis based on LSV data yields the value of i_0 as 3.25×10^{-7} $mA \cdot cm^{-2}$, with a Tafel slope of 116 $mV \cdot dec^{-1}$.

2.10. Supplementary Note 10

Demonstration of constant proton concentration in our experiments

Below, we present the numerical analysis that demonstrates a constant proton concentration in our experiments, following the reported procedure:[37,38]

In order to quantitatively evaluate the possible deviation of local pH near the electrode, here we define the concentration overpotential η_{conc} as reported:[37]

$$\eta_{conc} = \frac{RT}{F}\ln\left(\frac{[\mathrm{H}^+]_{\mathrm{surface}}}{[\mathrm{H}^+]_{\mathrm{bulk}}}\right) \tag{S1}$$

Here $[\mathrm{H}^+]_{\mathrm{surface}}$ and $[\mathrm{H}^+]_{\mathrm{bulk}}$ denote the proton concentrations near the surface and in the bulk, respectively. Based on this definition, the smaller the value of η_{conc}, a homogenous profile of pH in the solution is better satisfied.

Based on the derivation,[37] in phosphate buffer under a given electrochemical condition η_{conc} can be calculated as:

$$\eta_{\mathrm{conc}} = \frac{RT}{F}\ln\left(-\frac{10^{(\mathrm{pH}-\mathrm{pK_a})}\left(\lambda + 10^{(\mathrm{pH}-\mathrm{pK_a})}\lambda - 1\right)}{\lambda + 10^{(\mathrm{pH}-\mathrm{pK_a})}\lambda + 10^{(\mathrm{pH}-\mathrm{pK_a})}}\right) \tag{S2}$$

in which,

$$\lambda = \frac{i\delta}{FDC_{\mathrm{T}}} \tag{S3}$$

Here pH is the pH value of the bulk solution, $\mathrm{pK_a}$ the $\mathrm{pK_a}$ value for the buffer, *i* the current density of electrochemical reaction, δ the thickness of diffusion length, *D* the diffusion coefficient of phosphate buffer anions, and C_T the total concentration of the buffer in the solution. While the original derivation[37] assumes the reduction of proton with a positive value of *i*, these equations remain valid in our context of electrochemical ORR.

We calculated the values of η_{conc} by entering experimental parameters into the above equations. Here pH = 7.0, $\mathrm{pK_a} = 7.21$ for phosphate buffer[15], D = 1.27×10^{-9} $\mathrm{m^2\cdot sec^{-1}}$ for phosphate anions[39], $\delta = 70$ µm as shown in Supplementary Fig. 14 and Fig. 2f, and $C_{\mathrm{T}} = 12$ mM and 10 mM for the phosphate buffered saline (PBS) and minimal medium, respectively. The value of *i* within our experiments are *ca.* 80 $\mu\mathrm{A\cdot cm^{-2}}$ (Supplementary Fig. 16). Based on these values, we determine $\eta_{\mathrm{conc}} = -4$ mV and -5 mV in the PBS and minimal medium, respectively. These two values both correspond to a local pH of 7.1 in these scenarios, while the bulk pH of the solution is 7.0. The small difference between the local and bulk pH values support our claim of a minimal perturbation proton concentrations within the solution.

2.11. Supplementary Note 11

Rational of simulation model

The equation determining the local current density at the electrode surface is a concentration-dependent Tafel equation that takes into consideration the local concentration of redox species

in the proximity of the electrode. This equation and its Butler-Volmer variant can be found in the textbook “Electrochemical methods. Fundamentals and Applications” (2nd ed.) authored by A. J. Bard and L. R. Faulkner (section 3.4, for example). As stated in the textbook, such variants integrate the mass transport of limiting reagent in the redox reaction with electrochemical kinetics.

$$i_{loc} = -i_0 \frac{[O_2]}{c_{O_2}} \exp\left(\frac{-\alpha_c F\eta}{RT}\right) \tag{S4}$$

In the above equation, i_{loc} is the local current density of oxygen reduction reaction (ORR), i_0 the exchange current density, α_c = 0.5 the transfer coefficient, F Faraday constant, R gas constant, T the absolute temperature, $[O_2]$ the local O_2 concentration, and c_{O_2} the solubility of O_2 in water when equilibrated with air.

The anodic branch of the electrochemical reaction in numerical simulation is not necessary in practical simulation. In our work, Tafel equation (shown above) is an appropriate implication from the Butler-Volmer equation, when

$$\exp\left(\frac{-\alpha_c F\eta}{RT}\right) \gg \exp\left(\frac{(1-\alpha_c)F\eta}{RT}\right) \tag{S5}$$

Based on the textbook of Bard and Faulkner, the Tafel equation can be expected to hold when the back reaction, the anodic branch in our case, contributes less than 1% of the current, or

$$\exp\left(\frac{F\eta}{RT}\right) < 0.01 \tag{S6}$$

This implies $|\eta| > 118$ mV at 25 ℃ or $|\eta| > 120$ mV at 30 ℃ (microbial growth condition). The reaction of interests in our numerical simulation, oxygen reduction, has a thermodynamic redox potential of 1.23 V *vs*. RHE. The electrochemical potentials applied on the wire array in our experiments are no larger than 0.5 V *vs*. RHE. Therefore, the overpotential $|\eta|$ is large enough to justify the application of Tafel equation in numerical simulation.

Other species including H^+ and water are not considered in the simulation. The species participating ORR, including H^+ and water, are not limiting reagents and do not induce a significant change of local concentrations near the electrode, if any. Therefore, it is not necessary to include the mass transport of these species in the numerical simulation. Particular consideration is given to the mass transport of H^+. While the concentration of H^+ is low (pH = 7.0), the concentrations of phosphate buffer in the liquid medium, 12 mM and 10 mM for the PBS and minimal medium, respectively, are higher than the concentration of O_2, 0.246 mM in air.[40] This shows that the mass transport of proton is not the limiting factor, based on the

analysis following the procedure in previous reports.[37,38] A more detailed and quantitative analysis is provided in Supplementary Note 10.

We did not introduce Nernst-Planck equation for simulation given the high ionic strength and low current density involved in our experiments. Thanks to the high ionic strength of the liquid medium (170 mM and 50 mM for the PBS and minimal medium, respectively), the assumption of supporting electrolyte applies hence the migration term in the Nernst-Plank equation has minute contribution.

2.12. Supplementary Note 12

The effect of oxygen evolution reaction (OER) on the counter electrode to the validation of O_2 gradient in optical measurements

Our experimental results along with the fundamentals of fluidic dynamics suggest that the O_2 generated from the counter electrode does not interfere in our current study. As our microbial N_2 fixation occurs in a flow reactor with relatively slow flow rates (2 $mL \cdot min^{-1}$, linear velocity 3.3×10^{-2} $m \cdot s^{-1}$). Such small linear velocities of fluidic suggest that the liquid follows Newtonian fluidic and laminar flow patterns.[41] Since the counter electrode is on the opposite side of the flow channel (Supplementary Fig. 4), the O_2 generated from the counter electrode will be swept away by the fluidic without significantly impacting the pattern of O_2 concentrations near the wire array electrode (working electrode). The above argument is supported by our experimental data. Supplementary Fig. 14 displays the experimentally measured intensity (I_{em}) of fluorescent emission for $Ru(phen)_3^{2+}$, under the same setup that we mapped the microscopic O_2 gradient in Fig. 2. Cross-sectional mappings of I_{em} are displayed in the absence and presence of electrochemical potential (E_{appl}) in Supplementary Fig. 14a and Supplementary Fig. 14b, respectively. When E_{appl} = 0.5 V *vs*. RHE, the generation of O_2 on the counter electrode leads to decreased I_{em} near the counter electrode, yet such a change of I_{em} values cannot propagate through the channel as the I_{em} in the center of the channel remains the same between Supplementary Fig. 14a and Supplementary Fig. 14b. It indicates that the generation of O_2 on the counter electrode does not impact the O_2 gradient on the wire array thanks to the laminar flow of the fluid.

2.13. Supplementary Note 13

Characterization method of O_2 profile is not sensitive to the composition of the medium

Our experimental data suggest that our characterization method of O_2 profile is not sensitive to the composition of the medium. We conducted 1) the calibration of fluorescence lifetime versus O_2 concentration and 2) mapping of O_2 concentrations in the wire array electrode with the use of microbial growth medium (minimal medium) under the same condition as in PBS (Fig. 2f).

The calibration curves both show linear correlation between $[O_2]$ and $1/\tau$ in PBS and minimal medium, and the functions of linear fitting of the curves are almost identical ($[O_2]$/mM = $[1/(\tau/\text{msec})–4.56]/1.28$ for PBS and $[O_2]$/mM = $[1/(\tau/\text{msec})–4.50]/1.29$ for minimal medium). The $[O_2]$ profiles within wire array under the environment of both PBS and minimal medium are compared in Supplementary Fig. 15 (E_{appl} = 0.5 V *vs.* RHE). The results measured in minimal medium are not significantly different from the ones measured in PBS solution. Therefore, we conclude that our characterization method of O_2 profile is not sensitive to the composition of the medium within the range of O_2 concentrations measured in this work.

2.14. Supplementary Note 14

Association between microbes and wire array electrode

There are no specific driving forces such as an electrochemical one when the wire array electrode was initially exposed to microbial cultures. We also note that the gravity should not contribute to the microbial accumulation, because the whole setup, shown in Supplementary Fig. 4, was mounted on an inverted microscope and the wire array electrodes are indeed facing down (our illustration in the main figures are schematic and meant for the readers easy to understand). We postulate that the adhesion between the microbes and electrode should contribute to the initial retention of microbes within the wire array during the inoculation.

2.15. Supplementary Note 15

Biofilm formation during 120-hr incubation

Significant formation of biofilms was not found after 120-hr incubation of microbes in our nitrogen fixation experiments, based on the optical fluorescent images in Fig. 3c and 3f and data reported in main text. The calculated cell densities excluding the volume of wire arrays, 0.0067 cell·μm^{-3} for *X. autotrophicus* and 0.0045 cell·μm^{-3} for *B. japonicum*, are much lower than the 0.1 cell·μm^{-3} in common steady-state biofilms.[42,43]

3. Supplementary Tables:

3.1. **Supplementary Table 1: Comparison between N_2 fixation activity between inorganic | biological hybrid and natural root nodule systems**

Microbe	Plant	rN_m [a] mg·g^{-1}·hr^{-1}	rN_V [b] mg·L^{-1}·hr^{-1}	Ref
Xanthobacter autotrophicus	\	1.1 [d]	4.8	\
Bradyrhizobium japonicum	\	6.5 [d]	2.7	\
Bradyrhizobium japonicum	*Glycine max*	0.084	8.4 [c]	44
Bradyrhizobium japonicum	*Vigna angularis*	0.087	8.7 [c]	45
Rhizobium leguminosarum	*Pisum sativum*	0.018	1.8 [c]	46
Rhizobium leguminosarum	*Pisum sativum*	0.45	45 [c]	47
Rhizobium fredii	*Phaseolus vulgaris*	0.25	25 [c]	48
Azorhizobium caulinodans	*Sesbania rostrata*	0.87	87 [c]	49

[a] Total nitrogen fixed per unit dry weight of biomass in hybrid system or per unit dry weight of root nodule, one representative value is used for calculation if multiple data is presented in one reference. The average dry weight of biomass in our biologic | inorganic hybrid system throughout the N_2 fixation process is used for calculation.

[b] Total nitrogen fixed per unit volume of wire array or per unit volume of root nodule.

[c] Root nodule volume data not available in reference, assuming 90% water content and 1 g·mL^{-1} density in plant tissue.

[d] Assuming 15% N content in dry weight of microbes.

3.2. **Supplementary Table 2: Parameters used for model simulation**[40,50,51]

**Parameter name**	**Value**
Length of wire, h	50 μm
Diameter of wire, d	4 μm
Periodicity of wire array, p	10 μm, 15 μm, 30 μm, and 60 μm.
Diffusion layer thickness, d_D	20 μm
Diffusion coefficient of O_2, D_{O_2}	2.2×10^{-9} m²·s⁻¹
Bulk concentration of O_2, c_{O_2}	2.46×10^{-4} mol·L⁻¹
Equilibrium potential of O_2/H_2O, $E^0_{O_2/H_2O}$	1.23 V *vs.* RHE
Exchange current density, i_0 [a]	3.25×10^{-7} mA·cm⁻²
Cathodic charge transfer coefficient, α_c	0.5
Faraday constant, F	96485 C·mol⁻¹
The gas constant, R	8.314 J·mol⁻¹·K⁻¹
The temperature, T	298 K

[a] The exchange current density is measured via Tafel plot. See Supplementary discussion 2.4 for details.

3.3. **Supplementary Table 3: Phosphate-buffered saline (PBS)**

Component	Concentration ($g{\cdot}L^{-1}$)
NaCl	8
KCl	0.2
Na_2HPO_4	1.44
KH_2PO_4	0.24

The components were dissolved in 1 L DI water, pH adjusted to 7.0 and autoclaved under 121 ℃ for 30 min.

3.4. **Supplementary Table 4: Succinate nutrient broth**

**Component**	**Concentration (g·L^{-1})**
Nutrient broth	5.0
Yeast extract	4.0
NaCl	3.0
Sodium succinate·2 H_2O	6.3

The components were dissolved in 1 L DI water and autoclaved under 121 ℃ for 30 min.

3.5. **Supplementary Table 5: Minimal medium for *Xanthobacter autotrophicus***

**Component**	**Concentration (g·L^{-1})**
K_2HPO_4	1
KH_2PO_4	0.5
$NaHCO_3$	2
$MgSO_4 \cdot 7H_2O$	0.1
$CaSO_4$	0.032
$FeSO_4 \cdot 5\ H_2O$	0.01
trace elemental solution	1 mL L^{-1}

The components were dissolved in 1 L DI water, pH adjusted to 7.0 and autoclaved under 121 ℃ for 30 min. Ingredients for trace elemental solution is listed in Supplementary Table. 7.

3.6. **Supplementary Table 6: H-3 medium for *Bradyrhizobium japonicum***

**Component**	**Concentration (g·L^{-1})**
KH_2PO_4	2.3
$Na_2HPO_4{\cdot}7H_2O$	2.9
$MgSO_4$	0.5
$CaSO_4$	0.01
$MnCl_2$	0.005
$NaVO_3$	0.005
$FeSO_4$	0.05
trace elemental solution	5 mL L^{-1}

The components were dissolved in 1 L DI water and autoclaved under 121 ℃ for 30 min. Then the mixture was cooled down to 50 ℃. 5 mL filter-sterilized standard vitamin solution and 10 mL filter-sterilized $NaHCO_3$ (5%, w/w) were then added into the mixture. The solution pH was adjusted to 7.0. Ingredients for trace elemental solution are listed in Supplementary Table. 7. Ingredients for standard vitamin solution are listed in Supplementary Table. 8.

3.7. **Supplementary Table 7: Trace elemental solution**

**Component**	**Concentration (g·L^{-1})**
H_3BO_3	2.8
$MnSO_4·4H_2O$	2.1
$Na_2MoO_4·2H_2O$	0.75
$ZnSO_4·7H_2O$	0.24
$CuSO_4·5H_2O$	0.04
$NiCl_2·6H_2O$	0.12

The components were dissolved in 1 L DI water and autoclaved under 121 ℃ for 30 min. The solution is vigorously shaken and sonicated before use.

3.8. **Supplementary Table 8: Standard vitamin solution**

**Component**	**Concentration (g·L^{-1})**
Riboflavin	0.1
Thiamine-HCl	0.5
Nicotinic acid	0.5
Pyridoxine-HCl	0.5
Ca-pantothenate	0.5
Biotin	0.001
Folic acid	0.002
Vitamin B_{12}	0.01

The components were dissolved in 1 L DI water and filter-sterilized. The solution is stored under 4 ℃, and vigorously shaken and sonicated before use.

4. Supplementary Figures

4.1.

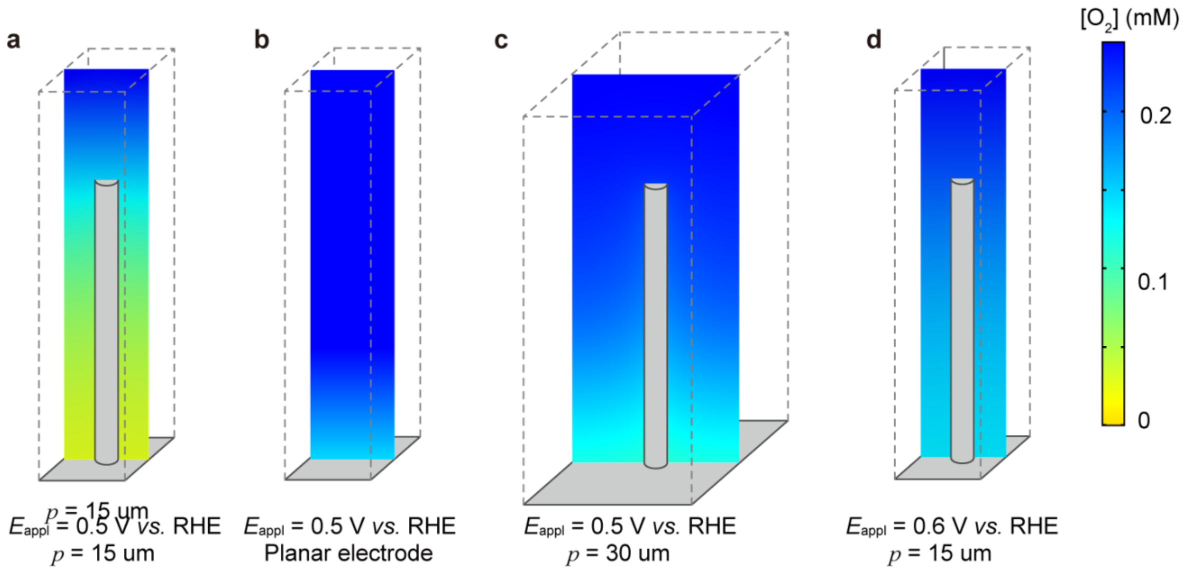

Supplementary Fig. 1: The simulated [O_2] profiles on planar and wire arrays electrodes. **a**, wire array p = 15 µm, E_{appl} = 0.5 V *vs.* RHE. **b**, planar electrode, E_{appl} = 0.5 V *vs.* RHE. **c**, p = 30 µm, E_{appl} = 0.5 V *vs.* RHE. **d**, p = 15 µm, E_{appl} = 0.6 V *vs.* RHE. In these models, l = 50 µm, d = 4 µm.

4.2.

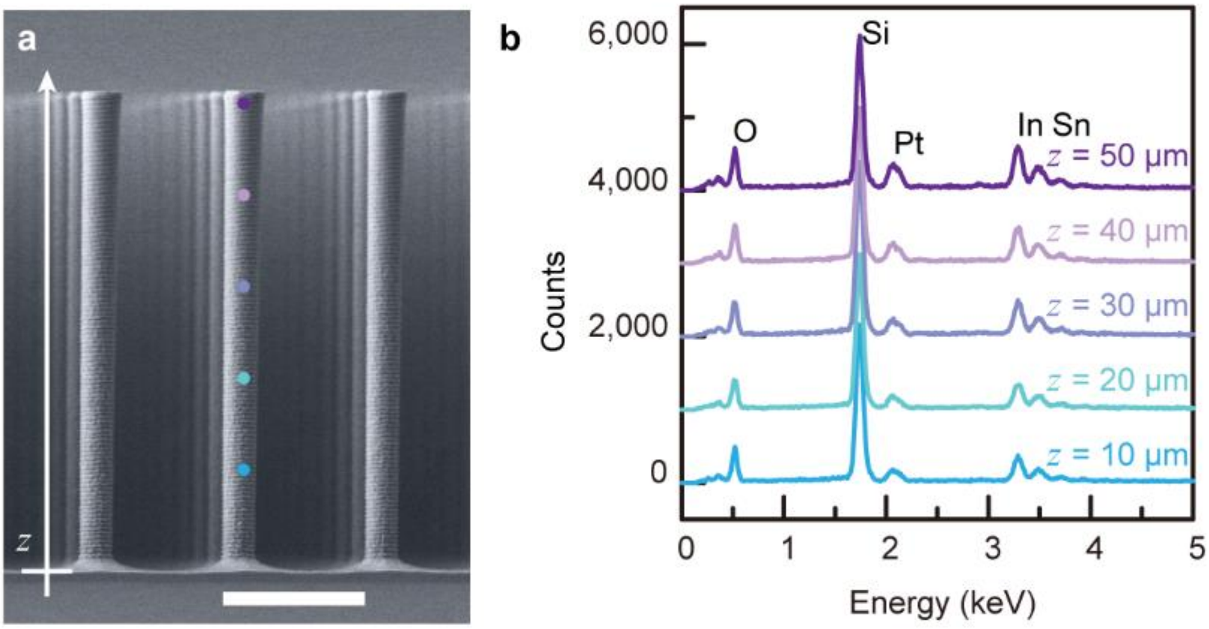

Supplementary Fig. 2: Morphological characterization of the prepared microwire array. **a**, cross-sectional SEM image depicting the side view of wire array. **b**, EDS spectra taken at depths highlighted in **a**. A homogenous distribution of Pt, In and Sn was observed along the prepared microwire array. Scale bar, 15 μm.

4.3.

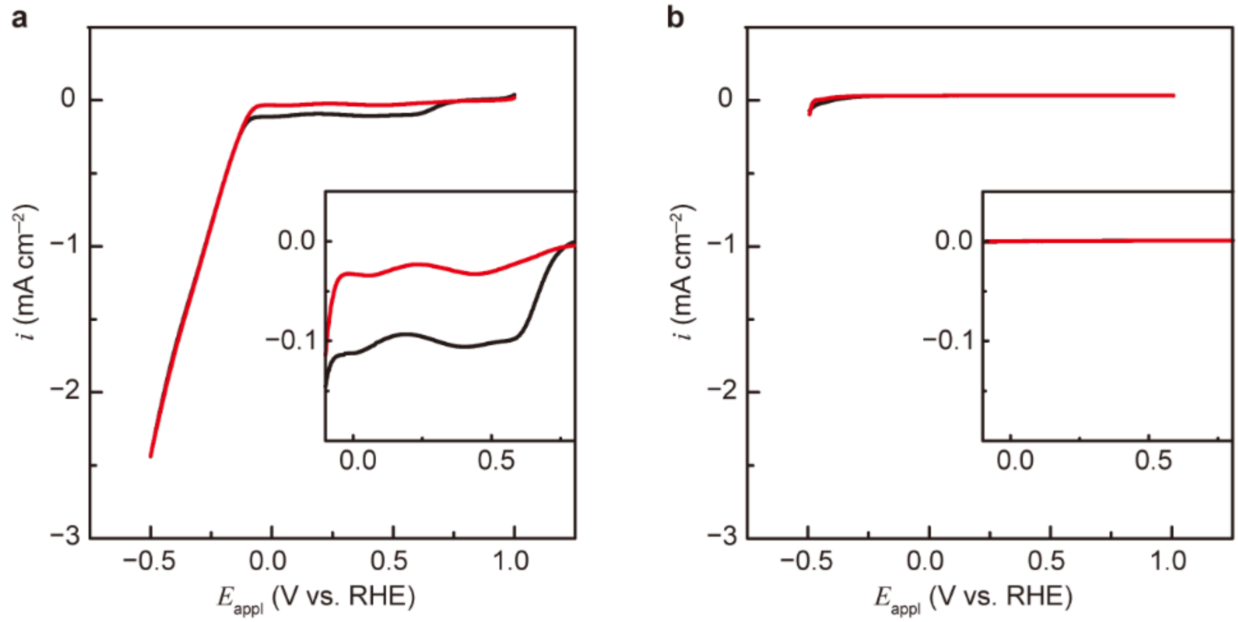

Supplementary Fig. 3: Linear scan voltammograms of wire arrays. a, with Pt deposition. **b**, without Pt deposition. Red, in Ar; black, in air. *i*, current density. Insets, detailed displays at the electrochemical window where oxygen reduction reaction takes place. The microwire array possesses the same morphologies as shown in Fig. 1c. 5 $mV{\cdot}s^{-1}$, minimal medium electrolyte (Supplementary Table 5), Ag paste as a pseudo-reference electrode (see Methods).

4.4.

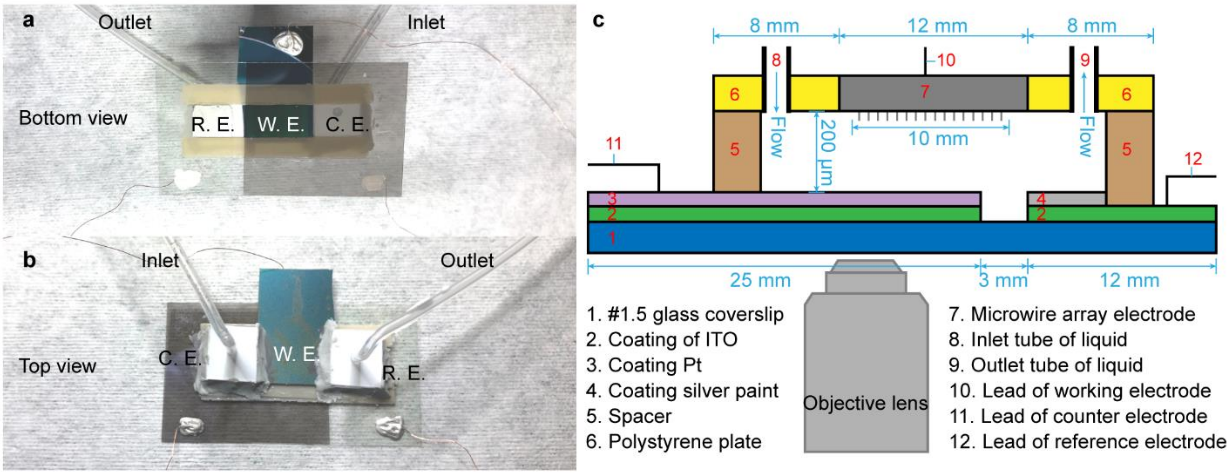

Supplementary Fig. 4: Photos of an electrochemical chamber for a confocal microscope. **a**, bottom view. **b**, top view. **c**, Cross-sectional scheme of electrochemical fluidic chamber used in the experiment described in Fig. 2 and Fig. 3. The objective lens shown in Fig. 2b is shown here in a setting of an inverted confocal laser scanning microscope. W. E., Si wire array working electrode. C. E., counter electrode. R. E., reference electrode.

4.5.

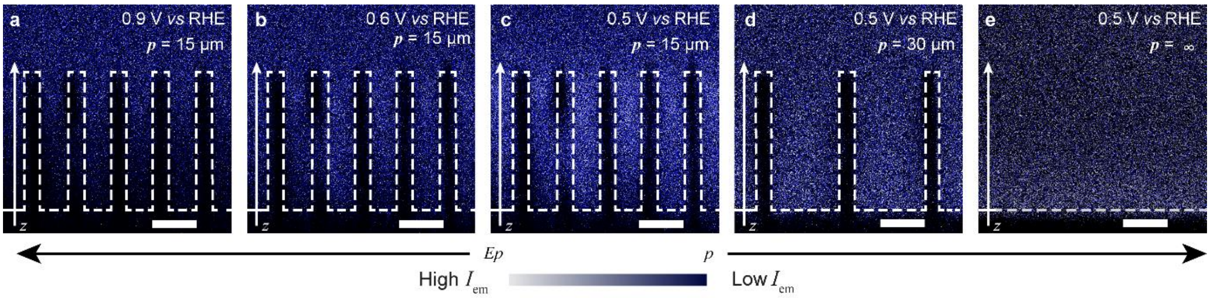

Supplementary Fig. 5: Cross-sectional mapping of O_2 gradients under different experimental conditions. The phosphorescence intensity of 0.1 mM of Ru(phen)$_3^{2+}$, pseudo-colored in blue, is a surrogate of local O_2 concentrations. Brighter color indicates higher intensity of phosphorescence emission, I_{em}. **a to c**, wire array shown in Fig. 1c, p = 15 µm, E_{appl} = 0.9 V (**a**), 0.6 V (**b**) and 0.5 V (**c**) *vs.* RHE. **d**, wire array with p = 30 µm, E_{appl} = 0.5 V *vs.* RHE. **e**, planar electrode, E_{appl} = 0.5 V *vs.* RHE. Scale bars, 15 µm.

4.6.

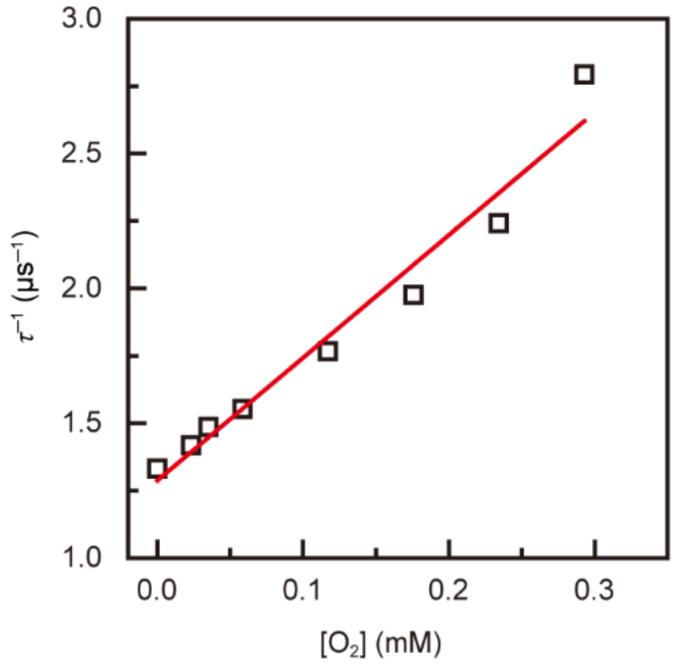

Supplementary Fig. 6: Plot of measured phosphorescence lifetime versus O_2 concentration. The phosphorescence lifetime of 0.1 mM $Ru(Phen)_3^{2+}$ PBS solution was determined at conditions of different O_2 concentrations in the electrochemical setup under a confocal microscope. This serves as the calibration curve for the quantification of O_2 concentrations.

4.7.

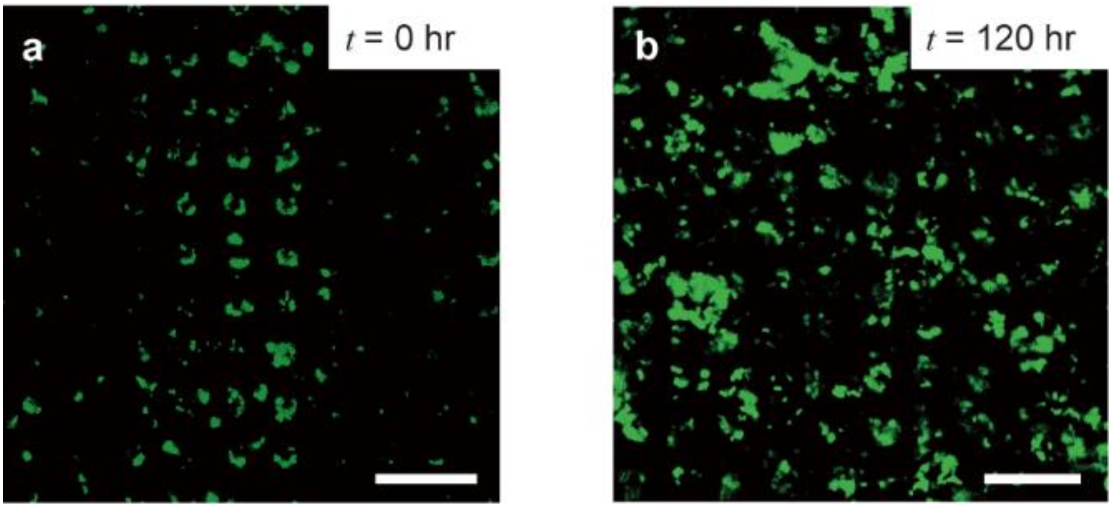

Supplementary Fig. 7: The increase of microbial population in wire array with N_2-fixing microbes. Fluorescence images of stained *X. autotrophicus* cells, pseudo-colored in green, before (**a**) and after (**b**) a 120-hr electricity-driven operation in the wire array shown in Fig. 1c. Images were taken at z = 20 μm. E_{appl} = −0.15 V *vs.* RHE. Scale bars, 30 μm.

4.8.

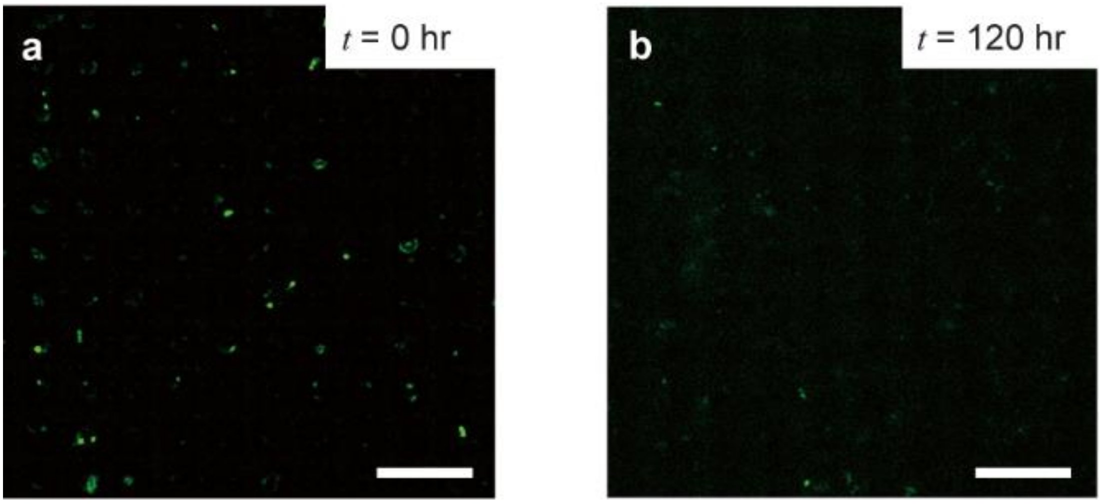

Supplementary Fig. 8: The stagnation of microbial population in wire array with non-N_2-fixing microbes. Fluorescence images of stained *B. japonicum-H1* cells, pseudo-colored in green, before (**a**) and after (**b**) a 120-hr electricity-driven operation in the wire array shown in Fig. 1c. Images were taken at z = 20 μm. E_{appl} = −0.15 V *vs*. RHE. Scale bars, 30 μm.

4.9.

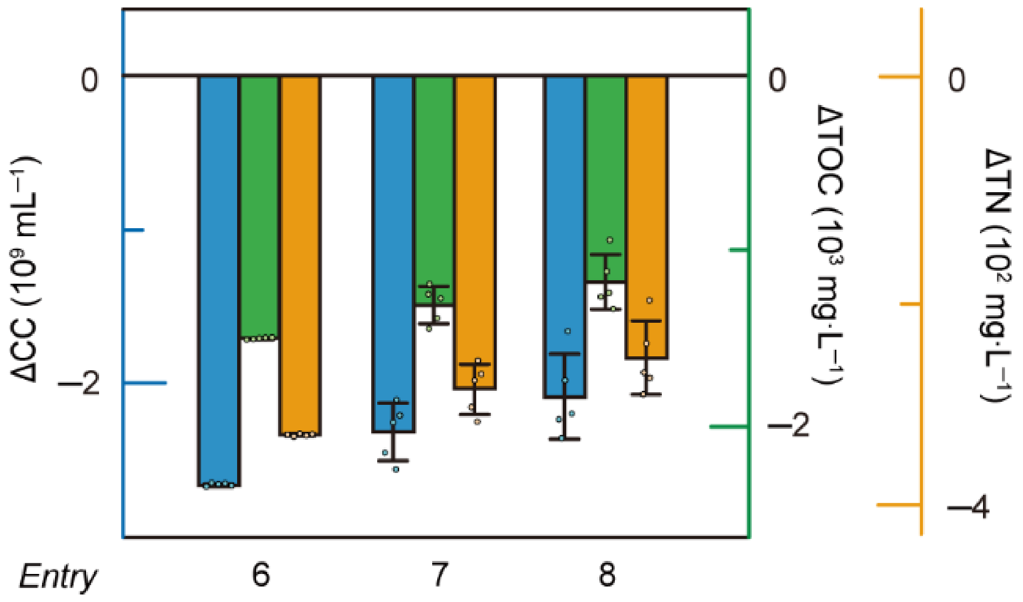

Supplementary Fig. 9: Changes of cell count (ΔCC), as well as the retained total nitrogen (ΔTN) and total organic carbon (ΔTOC) in biomass in the device for additional control experiments, calculated as the differences between values before and after 120-hr operation with the provision of electricity. The wire array design show in Fig. 1c were used in these experiments. Entry 6, no Pt deposition, E_{appl} = −0.15 V *vs.* RHE (n = 5). Entry 7, Pt deposited, E_{appl} = 0.05 V *vs.* RHE, a potential unable to generation H_2 and feed the microbes (n = 5). Entry 8, Pt deposited, in the absence of a E_{appl}. Microbes used in these experiments were *X. autotrophicus.* Individual measurement results are plotted as scattered dots. The bar graph plots the average and the error bars denote the standard deviation. Error bars = standard deviation (n = 5).

4.10.

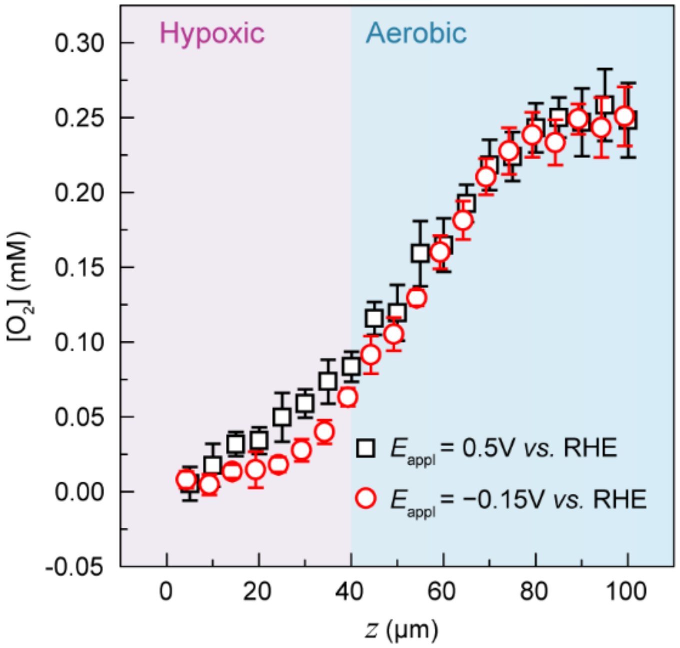

Supplementary Fig. 10: Profiles of O_2 concentrations inside and near the wire array electrode with E_{appl} = 0.5 V *vs.* RHE (black squares), and E_{appl} = −0.15 V *vs.* RHE (red circles) in minimal medium, both n = 3. Array periodicity p = 15 μm. The hypoxic (pink) and aerobic (blue) domains are suggested. Error bars = standard deviation (n = 3).

4.11.

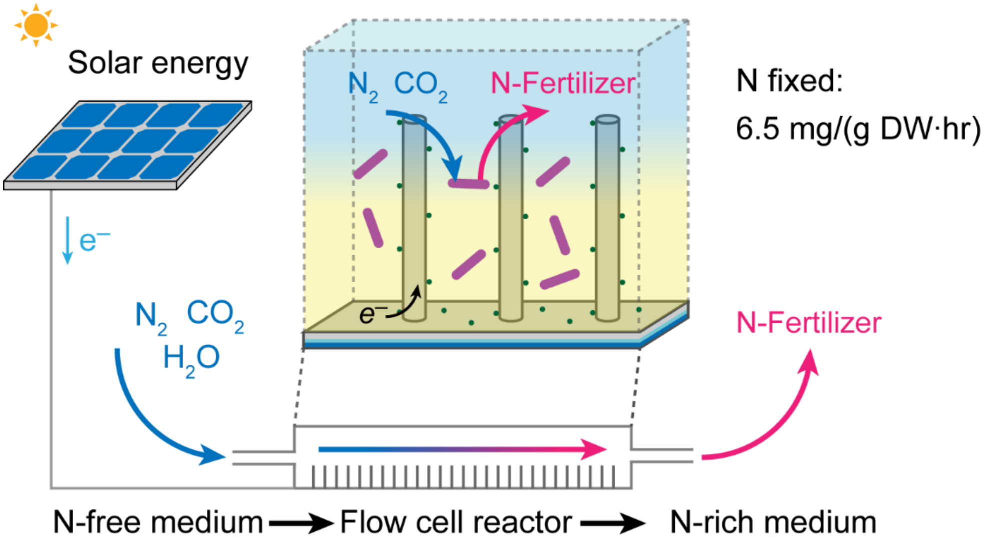

Supplementary Fig. 11: Schematic of the electricity-driven artificial root nodule that yields free-ammonia that can be potentially applied to crops. DW, dry weight of biomass in the reactor. The proposed device can be a standalone system in the farm. In such a system, renewable electricity will power the proposed platform, yielding an aqueous mixture of nitrogen fertilizer. The aqueous solution will be applied to the crop field subsequently.

4.12.

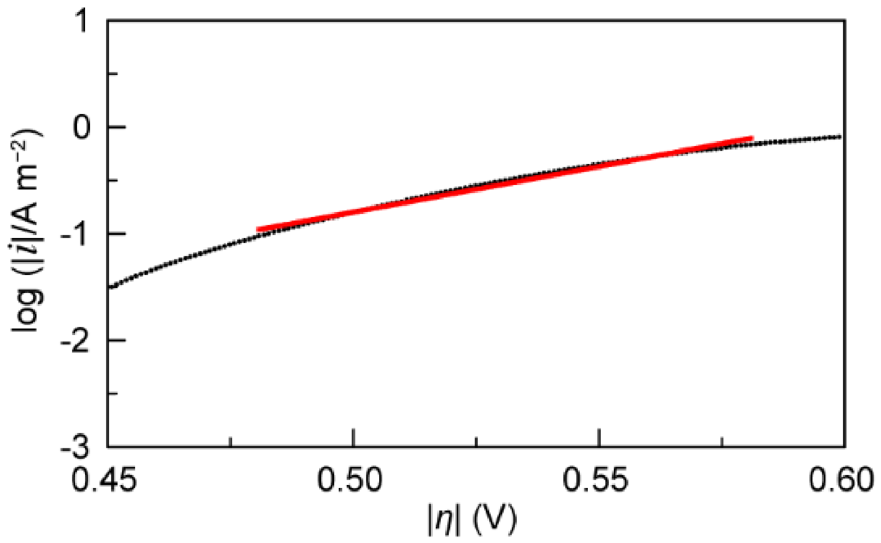

Supplementary Fig. 12: The Tafel plot: current density was logarithmically plotted against the absolute values of overpotential for oxygen reduction (E^0 = 1.23 V *vs.* RHE). Black, experimentally determined values using a Pt-sputtered wire array electrode. The Red, the fitted data based on the Tafel equation. The exchange current density (i_0) was found to be 3.25 $\times 10^{-7}$ mA·cm^{-2} with 116 mV·dec^{-1} as the value of Tafel slope.

4.13.

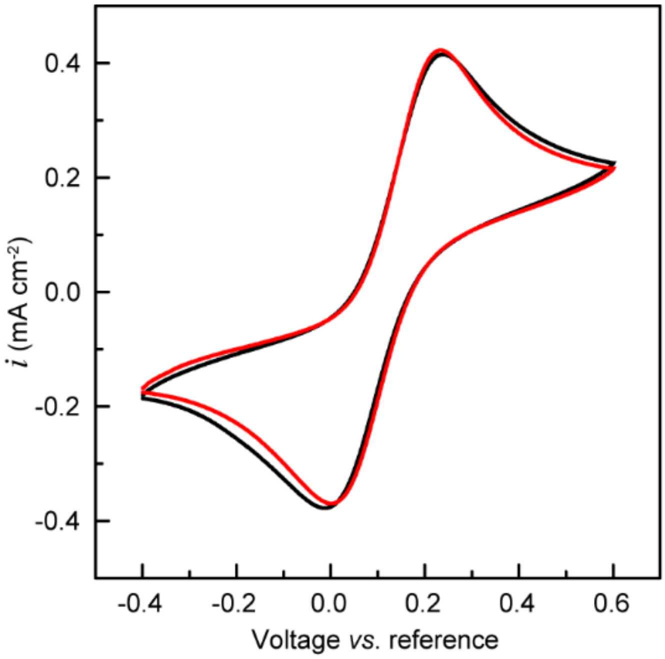

Supplementary Fig. 13: Cyclic voltammograms in ZoBell's solution using freshly made Ag pseudo-reference (black curve) and Ag pseudo-reference after 120-hr electrochemical operation in minimal medium (red curve). ZoBell's solution is used as the electrolyte, glassy carbon as working electrode, and Pt wire as counter electrode.

4.14.

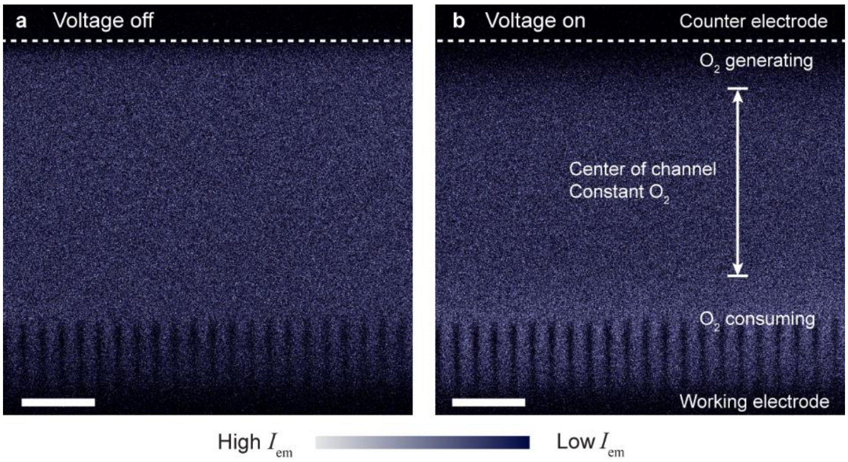

Supplementary Fig. 14: Cross-sectional I_{em} profiles in the absence (**a**) and presence (**b**) of E_{appl} = 0.5 V *vs.* RHE. The lower bound of the figure displays the microwire array (periodicity p = 15 µm) as the working electrode, while the dashed line at the top is the planar counter electrode. Scale bar, 60 µm. The images are pseudo-coloured blue. Brighter color indicates higher intensity of phosphorescence emission, I_{em}.

4.15.

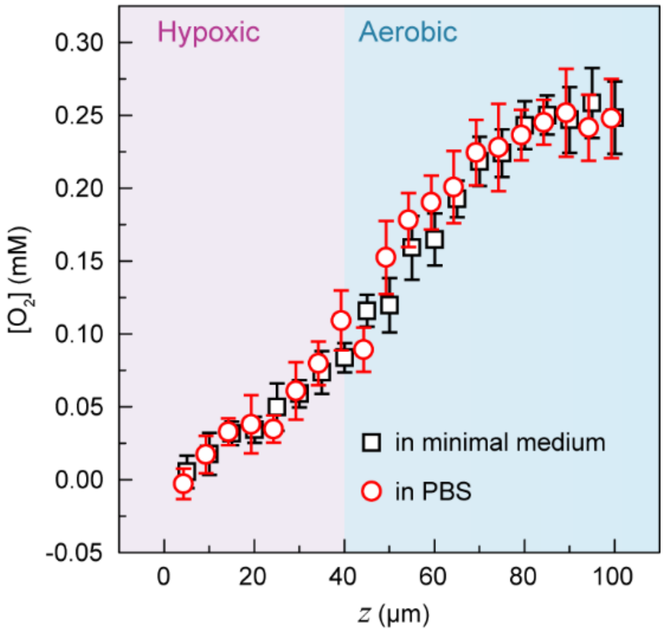

Supplementary Fig. 15: Profiles of O_2 concentrations inside and near the wire array electrode measured in PBS (red circles) and minimal medium (black squares). Both $n = 3$; p = 15 μm; E_{appl} = 0.5 V *vs*. RHE. The hypoxic (pink) and aerobic (blue) domains are suggested. The black trace is also presented as Supplementary Fig. 10; and the red trace is also presented as Fig. 2f. Error bars = standard deviation (n = 3).

4.16.

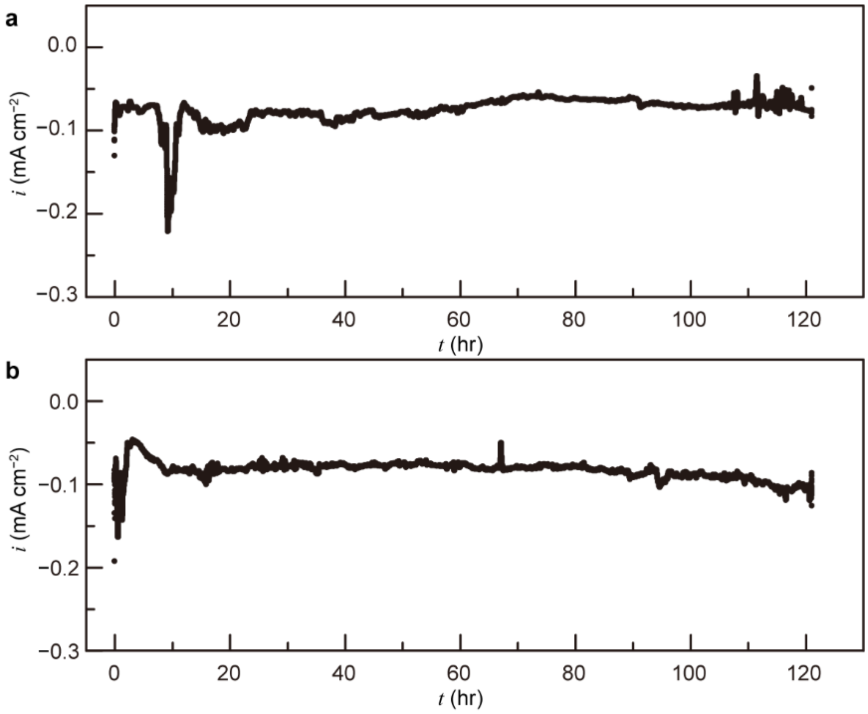

Supplementary Fig. 16: Current density in the designed hybrid N_2 fixation system in 120 hrs with *X. autotrophicus* (**a**) and *B. japonicum* (**b**) inoculated. Array periodicity p = 15 μm E_{appl} = −0.15 V *vs*. RHE.

4.17.

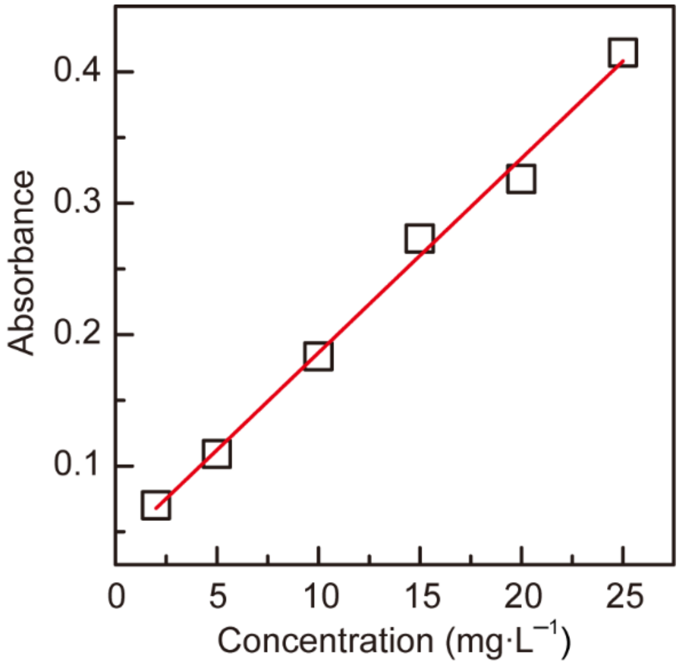

Supplementary Fig. 17: Calibration curve showing the correlation between the absorbance at 410 nm and nitrogen concentration in total nitrogen assay.

4.18.

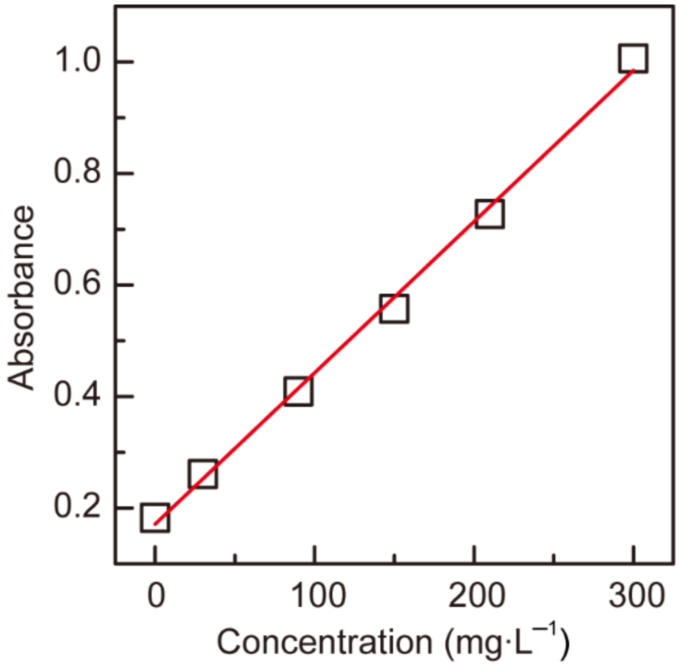

Supplementary Fig. 18: Calibration curve showing the correlation between the absorbance at 435 nm and organic carbon concentration in total organic carbon assay.

Reference

1 Ramey, B. E., Koutsoudis, M., Bodman, S. B. v. & Fuqua, C. Biofilm formation in plant–microbe associations. *Curr. Opin. Microbiol.* **7**, 602-609 (2004).

2 Rinaldo, S., Giardina, G., Mantoni, F., Paone, A. & Cutruzzolà, F. Beyond nitrogen metabolism: nitric oxide, cyclic-di-GMP and bacterial biofilms. *FEMS Microbiol. Lett.* **365**, fny029 (2018).

3 Wang, D., Xu, A., Elmerich, C. & Ma, L. Z. Biofilm formation enables free-living nitrogen-fixing rhizobacteria to fix nitrogen under aerobic conditions. *ISME J.* **11**, 1602-1613 (2017).

4 Rago, L. *et al.* Bioelectrochemical Nitrogen fixation (e-BNF): Electro-stimulation of enriched biofilm communities drives autotrophic nitrogen and carbon fixation. *Bioelectrochemistry* **125**, 105-115 (2019).

5 Rosche, B., Li, X. Z., Hauer, B., Schmid, A. & Buehler, K. Microbial biofilms: a concept for industrial catalysis? *Trends Biotechnol.* **27**, 636-643 (2009).

6 Dixon, R. & Kahn, D. Genetic regulation of biological nitrogen fixation. *Nat. Rev. Micro.* **2**, 621-631 (2004).

7 Glick, B. R. Plant Growth-Promoting Bacteria: Mechanisms and Applications. *Scientifica* **2012**, 15 (2012).

8 Wiegel, J. in *The Prokaryotes: Volume 5: Proteobacteria: Alpha and Beta Subclasses* 290-314 (Springer-Verlag, New York, 2006).

9 Malik, K. A. & Schlegel, H. G. Chemolithoautotrophic growth of bacteria able to grow under N_2-fixing conditions. *FEMS Microbiol. Lett.* **11**, 63-67 (1981).

10 Ferguson, B. J. *et al.* Molecular analysis of legume nodule development and autoregulation. *J. Integr. Plant Biol.* **52**, 61-76 (2010).

11 Fischinger, S. A., Drevon, J. J., Claassen, N. & Schulze, J. Nitrogen from senescing lower leaves of common bean is re-translocated to nodules and might be involved in a N-feedback regulation of nitrogen fixation. *J. Plant Physiol.* **163**, 987-995 (2006).

12 Liu, A., Contador, C. A., Fan, K. & Lam, H. M. Interaction and regulation of carbon, nitrogen, and phosphorus metabolisms in root nodules of legumes. *Front. Plant Sci.* **9**, 1860 (2018).

13 Blankenship, R. E. *et al.* Comparing photosynthetic and photovoltaic efficiencies and recognizing the potential for improvement. *Science* **332**, 805-809 (2011).

14 Minchin, F. R. & Witty, J. F. in *Plant Respiration: From Cell to Ecosystem* 195-205 (Springer Netherlands, 2005).

15 Rumble, J. R., Lide, D. R. & Bruno T. J. *CRC handbook of chemistry and physics : a ready-reference book of chemical and physical data.* 100th edition, Internet Version, (CRC Press, Boca Raton, 2019).

16 Guo, K., Prévoteau, A., Patil, S. A. & Rabaey, K. Engineering electrodes for microbial electrocatalysis. *Curr. Opin. Biotechnol.* **33**, 149-156 (2015).

17 Kumar, A. *et al.* The ins and outs of microorganism–electrode electron transfer reactions. *Nat. Rev. Chem.* **1**, 0024 (2017).

18 Nie, H. *et al.* Improved cathode for high efficient microbial-catalyzed reduction in microbial electrosynthesis cells. *Phys. Chem. Chem. Phys.* **15**, 14290-14294 (2013).

19 Zhang, T. *et al.* Improved cathode materials for microbial electrosynthesis. *Energy Environ. Sci.* **6**, 217-224 (2013).

20 Newman, J. S. & Tobias, C. W. Theoretical analysis of current distribution in porous electrodes. *J. Electrochem. Soc.* **109**, 1183-1191 (1962).

21 Yan, Y., Xia, B. Y., Zhao, B. & Wang, X. A review on noble-metal-free bifunctional heterogeneous catalysts for overall electrochemical water splitting. *J. Mater. Chem. A* **4**, 17587-17603 (2016).

22 Liu, B. *et al.* In situ electrodeposition of cobalt sulfide nanosheet arrays on carbon cloth as a highly efficient bifunctional electrocatalyst for oxygen evolution and reduction reactions. *ACS Appl. Mater. Interfaces* **10**, 30433-30440 (2018).

23 Lai, J. *et al.* Unprecedented metal-free 3D porous carbonaceous electrodes for full water splitting. *Energy Environ. Sci.* **9**, 1210-1214 (2016).

24 Sivanantham, A., Ganesan, P. & Shanmugam, S. Hierarchical $NiCo_2S_4$ nanowire arrays supported on Ni foam: an efficient and durable bifunctional electrocatalyst for oxygen and hydrogen evolution reactions. *Adv. Funct. Mater.* **26**, 4661-4672 (2016).

25 Zhu, Y. P., Liu, Y. P., Ren, T. Z. & Yuan, Z. Y. Self-supported cobalt phosphide mesoporous nanorod arrays: a flexible and bifunctional electrode for highly active electrocatalytic water reduction and oxidation. *Adv. Funct. Mater.* **25**, 7337-7347 (2015).

26 Shah, M. S. A. S. *et al.* Electrostatically regulated ternary-doped carbon foams with exposed active sites as metal-free oxygen reduction electrocatalysts. *Nanoscale* **10**, 19498-19508 (2018).

27 Xu, C. *et al.* Scalable conversion of CO_2 to N-doped carbon foam for efficient oxygen reduction reaction and lithium storage. *ACS Sustainable Chem. Eng.* **6**, 3358-3366 (2018).

28 Seh, Z. W. *et al.* Combining theory and experiment in electrocatalysis: insights into materials design. *Science* **355**, caad4998 (2017).

29 Liu, C., Colón, B. C., Ziesack, M., Silver, P. A. & Nocera, D. G. Water splitting-biosynthetic system with CO_2 reduction efficiencies exceeding photosynthesis. *Science* **352**, 1210-1213 (2016).

30 Ding, J., Ji, S., Wang, H., Pollet, B. G. & Wang, R. Mesoporous CoS/N-doped carbon as HER and ORR bifunctional electrocatalyst for water electrolyzers and zinc-air batteries. *ChemCatChem* **11**, 1026-1032 (2019).

31 Ganesan, P., Prabu, M., Sanetuntikul, J. & Shanmugam, S. Cobalt sulfide nanoparticles grown on nitrogen and sulfur codoped graphene oxide: an efficient electrocatalyst for oxygen reduction and evolution reactions. *ACS Catal.* **5**, 3625-3637 (2015).

32 Reece, S. Y. *et al.* Wireless solar water splitting using silicon-based semiconductors and earth-abundant catalysts. *Science* **334**, 645-648 (2011).

33 Sun, Y. J. *et al.* Electrodeposited cobalt-sulfide catalyst for electrochemical and photoelectrochemical hydrogen generation from water. *J. Am. Chem. Soc.* **135**, 17699-17702 (2013).

34 Esswein, A. J., Surendranath, Y., Reece, S. Y. & Nocera, D. G. Highly active cobalt phosphate and borate based oxygen evolving catalysts operating in neutral and natural waters. *Energy Environ. Sci.* **4**, 499-504 (2011).

35 Nichols, E. M. *et al.* Hybrid bioinorganic approach to solar-to-chemical conversion. *Proc. Natl. Acad. Sci. U. S. A* **112**, 11461-11466 (2015).

36 Esposito, D. V. *et al.* Low-cost hydrogen-evolution catalysts based on monolayer platinum on tungsten monocarbide substrates. *Angew. Chem. Int. Edit.* **49**, 9859-9862 (2010).

37 Jeremiasse, A. W., Hamelers, H. V. M., Kleijn, J. M. & Buisman, C. J. N. Use of biocompatible buffers to reduce the concentration overpotential for hydrogen evolution. *Environ. Sci. Technol.* **43**, 6882-6887 (2009).

38 Shinagawa, T. & Takanabe, K. Electrocatalytic hydrogen evolution under densely buffered neutral pH conditions. *J. Phys. Chem. C* **119**, 20453-20458 (2015).

39 Mihailescu, M. & Gabor, R. A. Diffusion coefficient of potassium dihydrogen phosphate using holographic interferometry. *Rom. J. Phys.* **56**, 399-410 (2011).

40 Kolev, N. I. in *Multiphase Flow Dynamics 4: Turbulence, Gas Adsorption and Release, Diesel Fuel Properties* 209-239 (Springer, Berlin Heidelberg, 2012).

41 Panton, R. L. *Incompressible Flow,* 4th edition, pp127-149, (John Wiley & Sons, Inc., Hoboken, New Jersey, 2013).

42 Koo, H. & Yamada, K. M. Dynamic cell–matrix interactions modulate microbial biofilm and tissue 3D microenvironments. *Curr. Opin. Cell Biol.* **42**, 102-112 (2016).

43 Drescher, K. *et al.* Architectural transitions in *Vibrio cholerae* biofilms at single-cell resolution. *Proc. Natl. Acad. Sci. U. S. A.* **113**, E2066-E2072 (2016).

44 Subramanian, P., Kim, K., Krishnamoorthy, R., Sundaram, S. & Sa, T. Endophytic bacteria improve nodule function and plant nitrogen in soybean on co-inoculation with *Bradyrhizobium japonicum* MN110. *Plant Growth Regul.* **76**, 327-332 (2015).

45 Delić-, D. *et al.* Nodulation and N_2 fixation effectiveness of *Bradyrhizobium* strains in symbiosis with adzuki bean, *Vigna angularis*. *Braz. Arch. Biol. Technol.* **53**, 293-299 (2010).

46 Fischinger, S. A. & Schulze, J. The importance of nodule CO_2 fixation for the efficiency of symbiotic nitrogen fixation in pea at vegetative growth and during pod formation. *J. Exp. Bot.* **61,** 2281-2291 (2010).

47 Lodwig, E. M. *et al.* Amino-acid cycling drives nitrogen fixation in the legume–*Rhizobium* symbiosis. *Nature* **422,** 722-726 (2003).

48 Sadowsky, M. J., Cregan, P. B. & Keyser, H. H. Nodulation and nitrogen fixation efficacy of *Rhizobium fredii* with *Phaseolus vulgaris* Genotypes. *Appl. Environ. Microb.* **54**, 1907-1910 (1988).

49 Ciccolella, C. O., Raynard, N. A., Mei, J. H-M., Church, D. C. & Ludwig, R. A. Symbiotic legume nodules employ both rhizobial *exo*- and *endo*- hydrogenases to recycle hydrogen produced by nitrogen fixation. *PLoS One* **5,** e12094 (2010).

50 Bard, A. J., Parsons, R., Jordan, J. *Standard Potentials in Aqueous Solution*. (Marcel Dekker, New York, 1985).

51 Ferrell, R. T. & Himmelblau, D. M. Diffusion coefficients of nitrogen and oxygen in water. *J. Chem. Eng. Data.* **12,** 111-115 (1967).
